# Signal Pathways Involved in the Interaction Between Tumor-Associated Macrophages/TAMs and Glioblastoma Cells

**DOI:** 10.3389/fonc.2022.822085

**Published:** 2022-05-04

**Authors:** Xiaojin Liu, Yuan Liu, Yiwei Qi, Yimin Huang, Feng Hu, Fangyong Dong, Kai Shu, Ting Lei

**Affiliations:** Sino-German Neuro-Oncology Molecular Laboratory, Department of Neurosurgery, Tongji Hospital, Tongji Medical College, Huazhong University of Science and Technology, Wuhan, China

**Keywords:** glioblastoma, macrophage, microglia, signal pathway, interaction, therapeutic targets

## Abstract

It is commonly recognized, that glioblastoma is a large complex composed of neoplastic and non-neoplastic cells. Tumor-associated macrophages account for the majority of tumor bulk and play pivotal roles in tumor proliferation, migration, invasion, and survival. There are sophisticated interactions between malignant cells and tumor associated-macrophages. Tumor cells release a variety of chemokines, cytokines, and growth factors that subsequently lead to the recruitment of TAMs, which in return released a plethora of factors to construct an immunosuppressive and tumor-supportive microenvironment. In this article, we have reviewed the biological characteristics of glioblastoma-associated macrophages and microglia, highlighting the emerging molecular targets and related signal pathways involved in the interaction between TAMs and glioblastoma cells, as well as the potential TAMs-associated therapeutic targets for glioblastoma.

## Introduction

Glioblastoma multiform (GBM) is the most common primary malignant tumor of the central nervous system with an annual incidence rate of 3-5/100,000 and a dismal prognosis of 14.6 months, accounting for about 50% of all gliomas (1, 2). Both intrinsic characteristics of cancer cells and extrinsic interaction with the sophisticated tumor microenvironment (TME) lead to treatment resistance and tumor aggression (3). TME comprises complex non-cell constituents, such as extracellular matrix, interstitial fluid, growth factors, cytokines, chemokines, and angiogenic molecules, and multicellular components including both immune and non-immune cells that form a tumor-supportive milieu in which tumor cells grow and infiltrate (4). The miscellaneous non-neoplastic cells closely interact with each other and neoplastic cells in the TME, contributing to strong interdependence that drives tumor aggression (5).

It has been largely demonstrated that glioma cells strongly interplay with the most abundant non-neoplastic immune infiltrates in the TME called tumor-associated macrophages (TAMs)/microglia (6, 7). Tumor-associated macrophages (TAMs) display remarkable diversity and plasticity in TME and can change their characteristics accordingly in response to environmental cues (8). Traditionally, TAMs are classified as two extreme polarizations with M1 polarization (classically activated macrophages) on one end and M2 polarization (alternatively activated macrophages) on another end, which is oversimplified in the context of GBM. A more informative macrophages classification leads to a spectrum of macrophage populations based on their function (9). Investigations revealed that macrophages with different phenotypes coexist within the same mouse and human TAM population. Generally, TAMs presented as a common theme of regulatory and immunosuppressive phenotype with high diversity (10).

Furthermore, TAMs account for 30-50% of GBM tumor bulk, so targeting TAMs may be a reasonable and promising adjunctive therapy for these difficult-to-control cancers (11). To fully understand the complex interaction between TAMs and glioblastoma cells, this paper reviews the biological characteristics of glioblastoma-associated macrophages and microglia, with emphasis on molecular targets and related signal pathways arising from the interaction between TAMs and glioblastoma cells, as well as the related potential therapeutic strategies for glioblastoma treatment.

## The Biology of Glioblastoma-Associated Macrophages and Microglia

**Figure 1:**
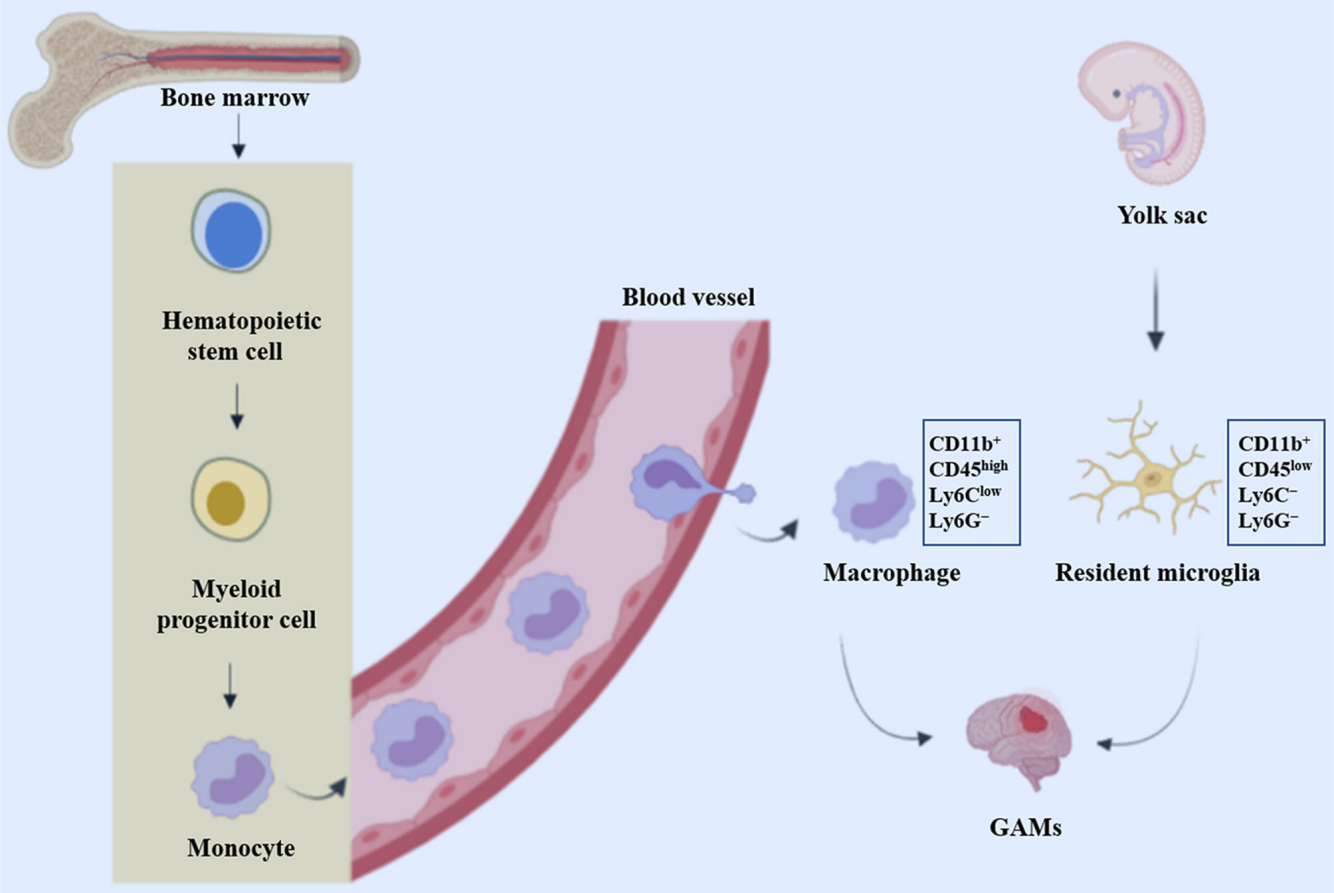
Origin of glioblastoma-associated macrophages and microglia. GAMs represent resident microglia and bone marrow-derived macrophages (BMDM), which originate from precursor cells in the yolk sac and bone marrow respectively.

TAMs are widely believed to represent two types of non-neoplastic immune cells that are similar in morphology and function but differ in the ontology: resident microglia and bone marrow-derived macrophages (BMDM) (12). Microglia are originated from myeloid precursors inhabited in the primitive yolk sac and are distributed throughout the brain during embryogenesis (13). These resident mononuclear cells function as key immune effector cells, playing pivotal roles in health and disease conditions of the central nervous system (CNS). In addition, other ontogenesis of brain microglia may reflect different waves of yolk sac hematopoiesis (14). Unlike resident microglia, macrophages typically penetrate through the blood-brain barrier into the CNS in the context of neuropathology, either through peripheral circulation or through direct channels connecting the skull bone and brain (15) (**Figure 1**).

Microglia are first discovered and described by Pio Del Rio Hortega about a century ago. Microglia ontogenesis and its homeostasis regulating mechanisms in health and disease conditions have been a hotspot for many decades (16). The main reasons for the confusion were the use of particular experimental systems, including chimera mice generated by bone marrow (BM) transplantation of lethally irradiated recipients, and monocyte classification schemes dependent on the expression of specific cell surface molecules (17). Through bone-marrow transplantation, researchers found that under homeostatic circumstances, a considerable proportion of microglia was superseded by donor-derived monocytes (18). Other similar studies have also indicated that both the endogenous microglia self-renewal and the dynamic recruitment of BM-derived microglial progenitors from the blood circulation contribute to augment in microglia density in reaction to CNS damage (19–23). Circulating progenitor cells contribute little to the brain microglia pool, suggesting that microglial proliferation during microgliosis (microglial activation) is mainly attributed to the local expansion of pre-existing resident microglia (24). These seemingly contradictory findings are eventually resolved with the use of chimeric animals produced by parabiosis, which does not necessitate either irradiation or transplantation. No microglia recruitment from the bloodstream was observed using two acute and chronic microglial activation (axotomy and neurodegeneration) (24). Additionally, Ajami et al. observed that acute peripheral monocytes recruitment in an experimental mouse model of autoimmune encephalitis (EAE) notwithstanding, these infiltrating cells vanished on remission and did not contribute to the endogenous microglia pool (25). Furthermore, recent fate-mapping studies have identified immature yolk sac progenitors as the predominant source of CNS microglia.

Taken together, these studies disclose that mouse myeloid progenitors from the blood circulation are not substantially participating in the pool of adult microglia after birth, thus determining that the pool of adult microglia mainly stems from yolk sac derived progenitors and maintain themselves by virtue of longevity and limited self-renewal (13, 24, 26). Single-cell RNA sequencing (scRNA-seq) of CD11b+ myeloid cells in naïve and GL261 glioma-bearing mice demonstrated considerable cellular and functional heterogeneity of myeloid cells in TME and is indicative of sex-specific discrepancies in responses of myeloid cells to gliomas (27). The ontogenesis of miscellaneous myeloid cells in the CNS is discussed in greater detail in a previous review (11). Furthermore, additional studies showed microglia located in different compartmentalization of mouse brain possess different transcriptomic information, suggesting that there are different microglia sub-phenotypes in both the human and mouse depending on their topological distribution and protein expression levels (28)

There is sufficient evidence that all tissue macrophages originate from a hematopoietic stem cell (HSC) pool during embryogenesis in the fetal liver (29–31). On day 12.5 of the embryogenesis (E12.5), HSCs develop into fetal monocytes characterized as two subsets including CCR2^+^Ly6C^+^CX3CR1^int^ and CCR2^+^Ly6C^−^CX3CR1^high^ (30, 32). Lineage tracing experiments showed that the Ly6C^+^ subset was an imperative precursor of the Ly6C^−^ subset with a restricted lifespan (29). In addition, the Ly6C^+^monocytic population emigrates from the fetal liver into the blood, leading to the downregulation of Ly6C and the initial expression of CX3CR1, which culminates in the tissue infiltrating macrophages (33). With few exceptions, splenocytes (34) and skin or gut macrophages remain in the tissue postnatally with longevity and limited self-renewal (31, 35, 36). After birth and during adulthood, hematopoiesis occurs mainly in the bone marrow, but also in the spleen, where Ly6C+monocytes are produced and extravasated from the bone marrow into the bloodstream by monocyte chemoattractant proteins (MCPs). Under healthy circumstances, monocytes have extremely short circulation half-lives with a period of 19 h for Ly6C^+^ and ~ 2.2 days for Ly6C^−^ (29). However, in the presence of pathological lesions such as brain tumors or inflammation, the blood-brain barrier (BBB) is disrupted, monocytes infiltrate and fill in the inflamed brain tissue, where they differentiate into BM-derived macrophages (BMDMs) (31, 37, 38).

In the monitoring mode, microglia are morphologically highly ramified and, when activated, they rapidly transform into an amoeboid shape (39). However, BMDM is morphologically similar to activated microglia and is indistinguishable on histological sections. When lineage tracing is not available, they can be discriminated employing differential expression of the CD11b/CD45 markers with CD45 low in microglia and high in macrophages, together with the Ly6C and Ly6G markers (CD11b^+^CD45^low^Ly6C^−^Ly6G^−^ for microglia and CD11b^+^CD45^high^Ly6C^low^Ly6G^−^ for macrophages) (40). In addition, emerging evidence support the view that microglia and macrophages are located in different regions of malignant gliomas, with macrophages appearing to be recruited early in tumorigenesis and to inhabit perivascular region (40). However, data on the dominant monocyte population in these tumors, with some studies demonstrating a microglia predominance (41), while others report infiltrating bone marrow-derived macrophages representing the majority of the glioma-associated macrophage (GAM) population (40, 42). These discrepancies could be attributed to specific experimental mouse model systems including the RCAS model and GL261 or T387 cell lines utilized in each of these studies, indicating that variations in GAM populations may be distinguishingly determined by the molecular characteristics of the glioma. In the future, the distinctive roles of microglia and blood monocytes in disease pathogenesis should be investigated thoroughly, to clarify the fate and origins of blood monocytes.

## Effects of Glioblastoma on TAMs

### Recruitment of TAMs

Glioblastoma cells recruit microglia and monocyte to evolve tumor niche through the establishment of chemokine gradients, resulting in the accumulation of TAMs in and around glioma tissue with an amoeboid morphology. Many factors mediate the recruitment of TAMs, such as chemokines, ligands of complement receptors, neurotransmitters, and ATP (43). It remains to be determined whether there exist distinct factors that attract intrinsic resident microglia or peripheral monocyte-derived macrophages to the tumor.

#### Classical Chemokine Signals

Monocyte chemoattractant protein-2 (CCL2) is the first chemoattractant factor to be discovered and CCL2/CCR2 signaling is significant in chemo-attraction during neuro-inflammatory processes (44). In some experimental glioblastoma models, tumor cells released CCL2 to attract macrophages (45), and CCL2/CCR2 blockade prolonged mouse survival (40, 41). Similarly, CCL2-expressing glioma cells produced a 10-fold increase in Ox42-positive cell density in rat models, while tumors overexpressing CCL2 increased more than three-fold, resulting in reduced rat survival (45). Moreover, Felsenstein et al. found that TAMs in human GBM specimens and syngeneic glioma model expressed CCR2 to various extents. Inoculating a CCR2-deficient strain for glioma model revealed a 30% reduction of TAMs intratumorally (46). Jung et al. revealed that Necrotic cells induced the expression of CCL2/MCP-1 and CCL20/MIP-3α in glioblastoma cells through activation of NF-κB and AP-1 and facilitated the recruitment of microglia into tumor tissues (47). However, Okada et al. observed a stronger correlation between MCP-3, rather than MCP-1 expression and the density of infiltrating microglia and macrophages, challenging to some extent the importance of MCP-1 to human glioma biology (48).

CX3CR1 is a receptor for the cytokine CX3CL1 (fractalkine), which is mainly expressed in microglia and is a reliable marker for microglia imaging *in vivo*. The CX3CL1 and CX3CR1 signaling cascade play pivotal roles in neuron-microglia communication, and downregulation of CX3CR1 compromises synapse plasticity during development (49). Nevertheless, there are inconsistent data concerning the importance of CX3CL1 in tumor-induced TAM infiltration (50–53). CSF-1/CSF-1R is another signal pathway involved in microglial recruitment. CSF-1 released from glioma cells functions as a chemo-attractant, and CSF-1R antagonist reduced the infiltration of TAMs and ameliorated glioblastoma invasion *in vivo* (54, 55). In addition, glioma cells also secrete hepatocyte growth factor (HGF) and scatter factor (SF) as chemo-attractants for microglia, but this has only been validated in a microglial cell line (56). CXCL12 is another potent chemokine for microglia and macrophage, especially recruiting TAMs toward hypoxic areas (57). The growth factor glial cell-derived neurotrophic factor (GDNF) was initially discovered as a secreting factor from the glial cell line B49, promoting the survival and differentiation of dopaminergic neurons. Mouse and human gliomas also secret GDNF, which function as a strong chemoattractant for microglia (58).

#### Emerging Chemokines and Molecules Involved

Recently, a growing number of emerging chemokines have been validated to be implicated in recruiting TAMs. For instance, Zhou et al. demonstrated that Glioblastoma stem cells (GSCs)-secreted periostin (POSTN) to recruit TAMs through the integrin αvβ₃, as blocking this signaling by an RGD peptide inhibited TAM recruitment. Silencing POSTN in GSCs markedly reduced TAMs infiltration, inhibited tumor growth, and prolonged survival of mice bearing GSC-derived xenografts (59). Osteopontin (OPN) is an effective chemokine for macrophages, which blocks the ability of glioma cells to recruit macrophages significantly. Integrin αv β5 (ITGαvβ5) is highly expressed on TAMs and constitutes a major OPN receptor. OPN deficiency in glioma cells led to a marked reduction in pro-tumor macrophages infiltrating the glioma (60). Profiling and functional studies in GBM models established that PTEN deficiency activates YAP1, which directly upregulates the expression of lysyl oxidase (LOX) expression. Mechanistically, secreted LOX induced TAMs recruitment *via* activation of the b1 integrin-PYK2 pathway in macrophages. LOX inhibition dramatically attenuated macrophage infiltration (61). Differentiated Glioblastoma Cells (DGCs) exhibited a significant augment in YAP/TAZ/TEAD activity compared with GSCs. The transcriptional target CCN1 of YAP/TAZ was released abundantly from DGCs, but not in GSCs, which promoted macrophage migration *in vitro* and macrophage infiltration into tumor niche *in vivo* (62). CLOCK and its heterodimeric partner BMAL1 prompted GSC self-renewal and triggered tumor-supportive immune response through transcriptional upregulation of OLFML3, a novel chemoattractant recruiting immune-suppressive TAMs into the TME. In GBM models, *CLOCK* or *OLFML3* depletion decreased intra-tumoral microglia density and extended overall survival (63).

Some emerging molecules recruit TAMs directly as chemokines,while others indirectly modify TAMs infiltration by modulating classical chemokine signals. Takenaka et al. reported that glioblastoma cells produced kynurenine to activate aryl hydrocarbon receptor (AHR) in TAMs, which promoted CCR2 expression, subsequently driving TAMs recruitment in response to CCL2 (64). An et al. demonstrated that EGFR and EGFRvIII cooperated to induce TAMs infiltration through KRAS-mediated upregulation of the chemokine CCL2 (65). By analyzing proteomic and transcriptional data available for GBM tumors from The Cancer Genome Atlas (TCGA), Lailler et al. manifested that GBM with high expression of phosphorylated ERK1/2 increased density of TAMs with a tumor-supportive M2 polarization. Using three human GBM cell lines in culture, they confirmed the existence of ERK1/2-dependent regulation of the production of CCL2 (66). Han et al. demonstrated that SETDB1 promoted AKT/mTOR-dependent CSF-1 induction and secretion, leading to macrophage recruitment in the tumor, and subsequently contributing to tumor growth (67). Additionally, De Boeck et al. found that IL-33 expression was positively correlated with the density of TAMs in a large subset of human glioma specimens and murine models, nuclear and secreted functions of IL-33 regulated chemokines that collectively recruited and activated circulating and resident innate immune cells. Conversely, loss of nuclear IL-33 crippled TAMs recruitment remarkably, inhibited glioma growth, and prolonged survival (68).

**Figure 2:**
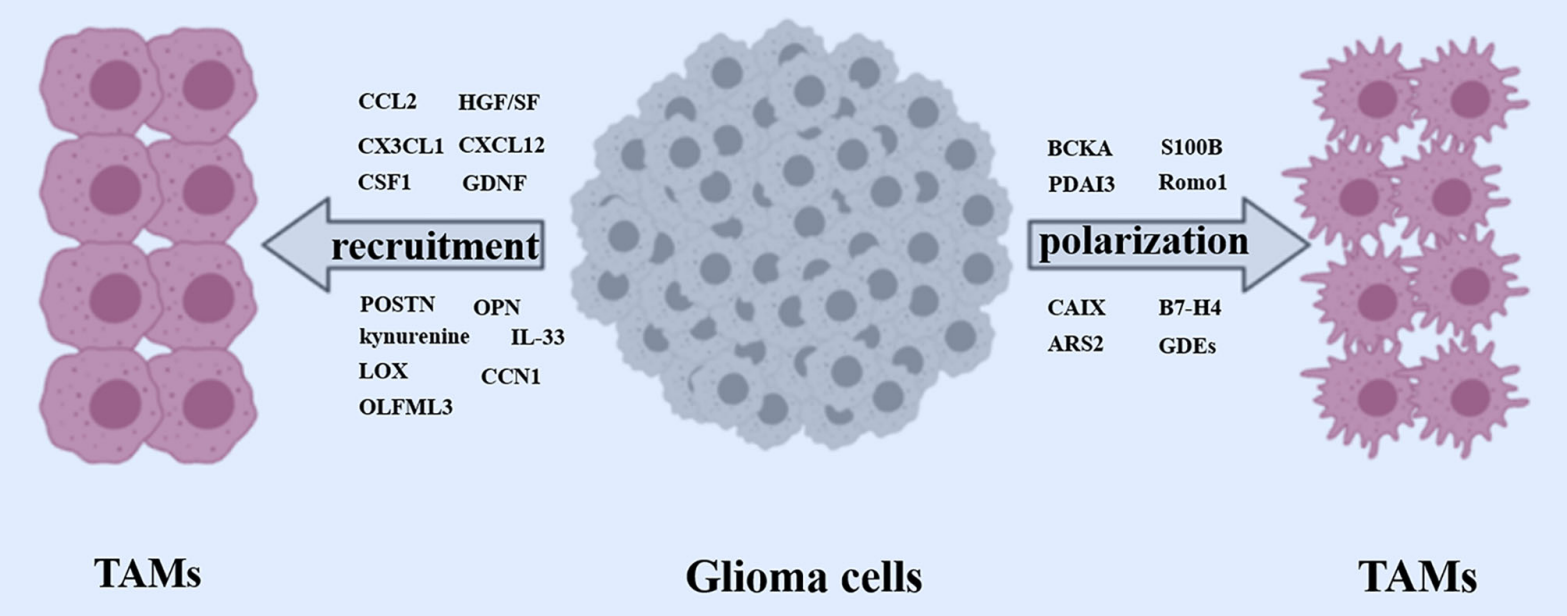
Recruitment and polarization of TAMs. Glioma cells released a wide array of factors (CCL2, CX3CL1, CSF1, GDNF, HGF/SF, CXCL12, POSTN, OPN, Kynurenine, LOX, IL-33, CCN1) to recruit TAMs. Meanwhile, some crucial glioma cells-derived factors (BCKA, PDAI3, S100B, Romo1, CAIX, B7-H4, ARS2, GDEs) are involved in polarizing TAMs toward a pro-tumor phenotype.

Transcriptome analysis indicated that most RSK1hi GBMs present as the mesenchymal subtype, and RSK1 expression was significantly associated with gene expression signature of immune infiltrates, especially in activated natural killer cells and M2 macrophages. In an independent cohort, Glaucia et al. confirmed that RSK1hi GBMs excluded long survivors, and RSK1 expression was positively associated with the protein level of the mesenchymal subtype marker lysosomal protein transmembrane 5, as well as with the TAM-associated CD68 (69). Tao et al. demonstrated that the Wnt-induced signaling protein 1 (WISP1) secreted by GSCs signals through Integrin α6β1-Akt to sustain M2 TAMs through a paracrine mechanism. Silencing WISP1 markedly disrupted GSC maintenance, reduced TAMs infiltration, and potently suppressed GBM growth (70). In conclusion, there are a variety of glioma-derived factors involved in TAMs infiltration toward the glioma (**Figure 2**). Digging novel key factors and the involved mechanism is still an attractive orientation moving forward in the future.

### Polarization of TAMs

TAMs are a heterogeneous population, not only because of their ontogenetic origin and distribution within the tumor but also to their functions. Historically, upon activation, TAMs were classified into two distinctive subsets, including M1 and M2 phenotype/polarization (71). Specifically, M1 is characterized by the classical activation of inflammatory receptors TLR2/4 and the secretion of pro-inflammatory cytokines such as TNF and IL-1β, polarized by lipopolysaccharide (LPS) either alone or in combination with Th1 cytokines such as IFN-γ and GM-CSF, with a pro-inflammatory phenotype. On the contrary. M2 is defined as the anti-inflammatory phenotype with the production of ARG1, IL-10, and IL-4, polarized by Th2 cytokines such as IL-4 and IL-13 (71, 72). TAMs are considered to resemble an M2 polarization in the context of GBM (73). Nevertheless, transcriptional analyses have shown that this dichotomous classification is an oversimplification of the otherwise sophisticated biology of these cells (74). Microglia and macrophages possess both M1 and M2 phenotypes in the setting of murine brain tumors (75). For instance, both IL-1β and ARG1 were found to be enriched in TAMs (40). In human GBM, TAMs more closely resemble the expression profile of non-polarized M0 macrophages (76).

There are various GBM-derived factors involved in the polarization of TAMs toward a pro-tumor M2-like phenotype. S100B, a member of the multigene family of Ca2^+^-binding proteins, is overexpressed by glioblastoma. Gao et al. demonstrated that low concentrations of S100B attenuated microglial activation through the induction of the STAT3 signal pathway (77). Glioblastoma-associated macrophages (GAMs) have a higher expression of ERp57/PDIA3 than in the microglia present in the surrounding parenchyma. Chiavari et al. demonstrated that reduced PDIA3 expression/activity in glioblastoma cells markedly limited the microglia pro-tumor polarization toward the M2 phenotype and the secretion of pro-inflammatory factors (78). Yin et al. demonstrated that arsenite-resistance protein (ARS2), a zinc finger protein directly activated the novel transcriptional target MGLL, encoding monoacylglycerol lipase (MAGL), which stimulate M2-like TAM polarization through the production of prostaglandin E2 (PGE2) (79). Under hypoxic conditions, the expression of CAIX (carbonic anhydrase IX) regulated through EGFR/STAT3/HIF-1α axis significantly increased in GBM, contributing to the polarization of tumor-associated monocytes/macrophages (TAM) toward a more tumor-supportive phenotype (80). Reactive oxygen species (ROS) modulator 1(Romo1) is highly expressed in macrophages and is associated with the poor prognosis of glioblastoma patients. using the glioblastoma murine model, Sun et al. found that the overexpression of Romo1 led to the M2 polarization of bone marrow-derived macrophages (BMDMs) through the mTORC1 signaling pathway (81).

TAMs acquire an immunosuppressive phenotype in the GBM microenvironment. Silva et al. showed that glioblastoma cells excreted large amounts of branched-chain ketoacids (BCKAs), metabolites of branched-chain amino acid (BCAA) catabolism. Tumor-excreted BCKAs can be taken up and re-aminated to BCAAs by TAMs. BCKAs exposure attenuated the phagocytic activity of macrophages (82). In both *in vitro* and *in vivo* GBM mouse models, GBM-initiating cells induced mTOR signaling in the microglia but not bone marrow-derived macrophages. mTOR-mediated regulation of STAT3 and NF-κB activity promoted an immunosuppressive microglial phenotype, which hindered effector T-cell infiltration, proliferation, and immune reactivity, thereby contributing to tumor immune evasion and tumor aggression (83). Yao et al. identified that B7-H4+ glioma infiltrated macrophages/microglia showed immunosuppressive phenotype which could be regulated by IL-6 excretion. IL-6-activated STAT3 bound to the promoter of the B7-H4 gene and enhanced B7-H4 expression on TAMs, resulting in an immunosuppressive phenotype of TAMs, which contributed to GBM progression (84).

The components of tumor-derived exosomes such as microRNAs and proteins induce macrophages to M2-like polarization to support tumor growth (85, 86). Xu et al. found that compared with normoxic glioma-derived exosomes (N-GDEs), hypoxic glioma-derived exosomes (H-GDEs) drastically facilitated autophagy and M2-like macrophage polarization, which subsequently promoted glioma proliferation and migration *in vitro* and *in vivo*. The interleukin 6 (IL-6) and miR-155-3p were highly expressed in H-GDEs. Further experiments showed that IL-6 and miR-155-3p induced M2-like macrophage polarization through the IL-6-pSTAT3-miR-155-3p-autophagy-pSTAT3 positive feedback loop, contributing to glioma progression (87). The Glioblastoma-derived exosomes (GDEs) traversed the monocyte cytoplasm, resulting in a reorganization of the actin cytoskeleton, and skewed monocytes toward the immune suppressive M2 phenotype, including programmed death-ligand 1 (PD-L1) expression. Mass spectrometry analysis demonstrated that the GDEs contain a variety of contents, including members involved in the signal transducer and activator of transcription 3 (STAT3) pathway that functionally mediate this pro-tumor immune-suppressive switch (88).

In addition to genetic regulation, a scenario of distinct histone modifications was identified to underlie the polarization of microglia by glioma, which demonstrates the contribution of epigenetic mechanisms to glioma-induced “transcriptional memory” in TAMs resulting in the tumor-supportive phenotype (89). Altogether, GAMs are genetically and epigenetically educated by a variety of factors from within glioblastoma cells or GDEs, leading to a pro-tumor immunosuppressive polarization, which results in GBM progression (**Figure 2**).

### Chemoradiotherapy and TAMs

The impacts of conventional therapies on TME have been largely investigated, indicating that chemoradiotherapies not only exert a direct cytotoxic effect on tumor cells but also modulate the immune infiltrates either in an anti-tumor or pro-tumor direction, depending on tumor types and chemotherapeutic agents (90–92). Chemoradiotherapy has a huge impact on TAMs recruitment and polarization. A clinical microdialysis study demonstrated that radiotherapy induced an immediate inflammatory reaction leading to TAMs recruitment, which was correlated with a short survival time in malignant glioma (93). Irradiation leads to the alteration of multiple pathways in the context of GBM. Particularly, it modifies the macrophage polarization, rendering them more supportive of tumor growth (91). Although controversies exist, mainstreams reported that chemotherapy induced TAMs recruitment and programmed them toward an immunosuppressive tumor-supportive polarization, contributing to tumor angiogenesis, T cell immunity suppression, and activating anti-apoptotic programs in cancer cells to induce chemoresistance (90, 94). Therefore, incorporating TAMs-targeting therapy into chemoradiotherapy may provide a promising choice for GBM treatment.

### Metabolic Reprogramming of TAMs

TAMs are characterized by remarkable plasticity and dynamic metabolic trait (95). In reaction to the altered metabolic profile of TME, TAMs evolve toward a cellular state which prioritizes utilizing glycolysis, fatty acid synthesis (FAS), and glutamine-glutamate metabolism (96, 97), influencing TAMs recruitment and polarization. Reciprocally, these functionally reprogrammed TAMs secret a wide range of altered cytokines and angiogenic factors contributing to tumor growth and survival (98–100). Won et al. elucidated in review (101) that loco-regional metabolic signals released from tumor environments (glucose, glutamine, cystéine, lactate, IDO, adenosine, itaconic acid, acidic pH) have a huge impact on the polarization fate and immunosuppressive functions of TAMs, thus possibly leading to immune tolerance and treatment resistance in GBM. Therefore, regulation of the promoters and enhancers of tolerized genes involved in metabolism and lipid biosynthesis may reverse the immune tolerance, transcriptionally rewiring the intracellular signaling of innate immune cells to make macrophages more competent in response to stimulation (10). Similarly, Carroll et al. found that inhibition of fatty acid synthase, which catalyzed the synthesis of long-chain fatty acids, prevented the proinflammatory response in macrophages (102). Intriguingly, metabolic profiling showed that exposure to b-amyloid stimulated acute reactive microglial inflammation accompanied by metabolic reprogramming from oxidative phosphorylation to glycolysis. Moreover. metabolic strengthening with recombinant interferon-γ treatment counteracted the defective glycolytic metabolism and inflammatory functions of microglia (103). Such microglial metabolic switch may also exert huge influences on GBM development.

## TAMs Remodeling GBM Proliferation and Invasion

The fact that a great number of TAMs accumulated in and around glioma bulk has intrigued the investigators to explore their roles in tumor proliferation, migration, and invasion. As expected, accumulating evidence indicated that TAMs promote glioma growth and invasion *in vitro* and *in vivo*. One study has noted long before that the motility of the murine glioma cells was increased threefold at the presence of microglial cells *in vitro*. By contrast, endothelial cells and oligodendrocytes only slightly promoted glioma motility (104). *In situ*, organotypic brain slices can be used to monitor glioma growth. these slices showed reduced invasion and growth of gliomas When microglia cells were removed with liposomes filled with the toxin clodronate (105). In addition, an alternative *in vivo* approach made the use of transgenic mice expressing the herpes simplex virus thymidine kinase gene under the control of the CD11b promoter, which was specifically expressed by microglia in the central nervous system. When ganciclovir was infused into the brain, there was a prominent reduction in microglia number, concomitantly resulting in attenuated glioma growth *in vivo* (106).

### Cytokine Signaling

**Figure 3:**
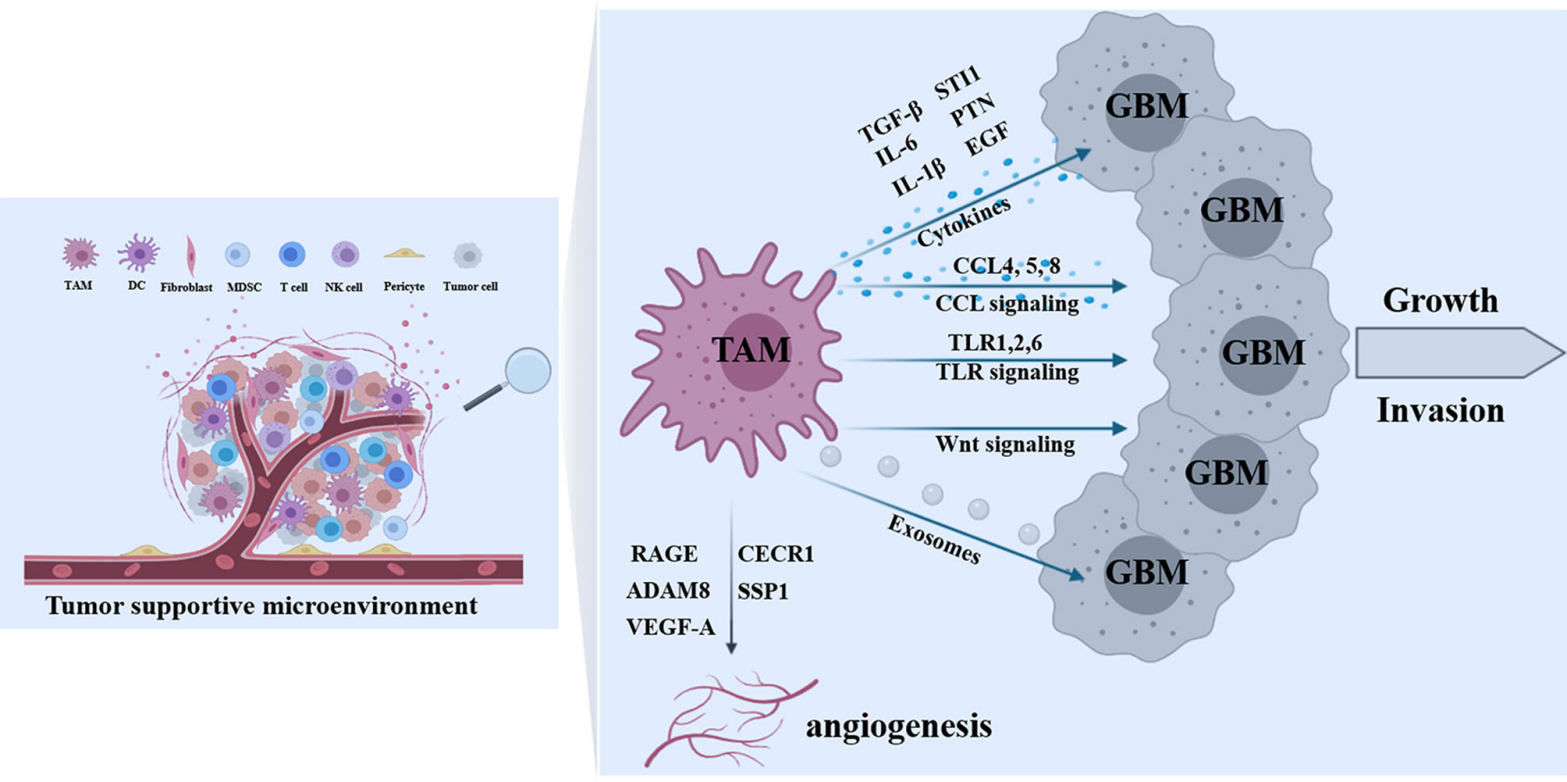
Effects of TAMs on tumor progression. In the tumor-supportive microenvironment of GBM, a variety of TAMs-derived factors contribute to tumor growth and invasion including cytokines (TGF-β, IL-6, IL-1β, STI1, PTN, and EGF), molecules in CCL signaling (CCL4, 5 and 8), proteins in TLR signaling (TLR1,2 and 6), Wnt signal cascades, and TAM-derived exosomes. In addition, some TAMs-derived molecules (RAGE, ADAM8, CECR1, SSP1, and VEGF-A) are implicated in tumor angiogenesis.

As mentioned above, there are a variety of factors from glioblastoma cells to induce TAMs recruitment and polarization. Meanwhile, various factors from TAMs have been reported to promote glioma proliferation, migration, and invasion (**Figure 3**). CCL2 released from glioma is a critical chemokine for TAMs and simultaneously triggers IL-6 release from microglia, thereby promoting the invasiveness of glioma cells (107). IL-6 secreted by *in situ* macrophages regulated the direction of a PGK1-catalyzed reaction by increasing PDPK1-dependent PGK1 phosphorylation in glioblastoma cells, promoting glycolysis and proliferation of tumor cells (108). Lu et al. demonstrated that interleukin 1β (IL-1β) produced by M2 macrophages activated phosphorylation of the glycolytic enzyme glycerol-3-phosphate dehydrogenase (GPD2) at threonine 10 (GPD2 pT10) through phosphatidylinositol-3-kinase-mediated activation of protein kinase-delta (PKCδ) in glioma cells. Blocking IL-1β generated by macrophages or Inhibition of PKCδ or GPD2 pT10 in glioma cells attenuated the glycolytic rate and proliferation of glioma cells (109). In addition, microglia synthesized and released stress-inducible protein 1 (STI1), a cellular prion protein-ligand that increased the proliferation and migration of glioblastomas *in vitro* and *in vivo* (110). Microglia release epidermal growth factor (EGF), which also stimulates glioblastoma cell invasion (54). Transforming growth factor-β (TGF-β) is predominantly produced by microglia when studied in co-culture systems, and blocking the TGF-β function impairs glioma growth (111). In addition, TGF-β2 induced the expression of matrix metalloprotease-2 (MMP2) and suppressed the expression of tissue inhibitors of metalloproteinases (TIMP)-2, which degraded the extracellular matrix to promote glioma invasion (112). Targeting TGF-β signaling was initially considered as a potential anti-tumor therapy, However, systemic inhibition of TGF-β signaling led to acute inflammation and disturbance of immune system homeostasis (111). CECR1 is a potent regulator of TAM polarization and is consistently highly expressed by M2-type TAMs, particularly in high-grade glioma. CECR1 mediated paracrine effects in M2-like TAMs stimulated MAPK signaling and activated the proliferation and migration of glioma cells (113). Shi et al. found that TAMs secreted abundant pleiotrophin (PTN) to stimulate glioma stem cells (GSCs), thus promoting GBM malignant growth through PTN-PTPRZ1 paracrine signaling. Co-implantation of M2-like macrophages (MLCs) promoted GSC-driven tumor growth, but depletion of PTN expression in MLCs mitigated their pro-tumorigenic activity. Disrupting PTPRZ1 abrogated GSC maintenance and tumorigenic potential. Moreover, Interference of PTN–PTPRZ1 signaling by shRNA or anti-PTPRZ1 antibody potently suppressed GBM tumor growth and prolonged animal survival (114).

### CCL/CCR Axis

Furthermore, the chemokine (C-C motif) ligand is an important cluster of molecules involved in the process of TAMs-mediated glioma progression. Wang et al. found that both hypoxia and macrophage supernatant promoted GBM cells invasion and matrix metalloproteinase (MMP)-9 expression, and hypoxia modulated the invasive activity of GBM cells by upregulating CCR5 expression. The supernatant of hypoxic macrophages also showed a greater pro-invasion effect than that of normoxic macrophages by increasing CCL4 secretion. Moreover, they found that interferon regulatory factor-8 (IRF-8) was possibly involved in hypoxia-modulated CCL4 expression of macrophages. Taken together, the study found that the CCL4-CCR5 axis played significant roles in TAM-mediated glioblastoma invasion, and hypoxia enhanced the interaction between these two types of cells by upregulating both CCL4 and CCR5 expression, respectively (115). chemokine (C-C motif) ligand 5 (CCL5) was reported to modulate the migratory and invasive activities of human glioma cells in association with MMP2 expression. In response to CCL5, glioma cells synchronously upregulated intracellular calcium levels and p-CaMKII and p-Akt expression levels. Inhibition of p-CaMKII suppressed CCL5-mediated glioma invasion and upregulation of MMP2. Glioma cells tended to migrate toward GAM-conditioned media activated by the granulocyte-macrophage colony-stimulating factor (GM-CSF) in which CCL5 was abundant. This homing effect was related to MMP2 upregulation and could be ameliorated either by controlling intracellular and extracellular calcium levels or by CCL5 antagonism (116). In addition, CCL8 was highly expressed by TAMs and contributed to pseudopodia formation by GBM cells. CCL8 dramatically activated ERK1/2 phosphorylation in GBM cells and promoted invasion and stemlike traits of GBM cells through CCR1 and CCR5. Blocking TAM-secreted CCL8 by neutralized antibody markedly attenuated invasion of glioma cells (117).

### TLR Signal Pathways

Toll-like receptors are prominent detectors of DNA fragments or bacterial cell wall components and are crucial for mediating immunologic responses to pathogens (118). TLRs signaling pathways play an important role in the interaction between microglia and glioma, among which TLR2 is considered to be the main TLR that triggers MT1-MMP upregulation in microglia. Therefore, the implantation of mouse GL261 glioma cells into TLR2 knockout mice resulted in markedly smaller tumor volume and better survival rates compared with wild-type control mice. TLR2 forms heterodimers with TLR1 and TLR6, which was critical for modulating MT1-MMP expression, while silencing of both TLR1 and TLR6 resulted in reduced MT1-MMP expression. In addition, treatment with TLR2-neutralizing antibodies reduced glioma-induced microglial MT1-MMP expression and attenuated glioma growth (119). In a screen for endogenous ligands secreted from glioma cells, versican was identified as a candidate molecule for triggering TLR2 signaling cascade (120). Versican exists as different splice variants such as V0, V1, and V2. The V0 and V1 isoforms are highly expressed in mouse and human gliomas and decreased glioma versican expression is correlated with reduced microglial MT1-MMP expression *in vitro* and *in vivo*. Furthermore, implantation of versican silenced glioma cells resulted in smaller tumors and longer survival rates relative to controls. Remarkably, the effect of versican signaling on glioma growth was reliant on the presence of microglia. Versican-mediated TLR2 expression polarized microglia into a pro-tumorigenic phenotype featured by the upregulation of MT1-MMP and MMP9 expression. This feed-forward loop presented us with a great example of the interdependent microglia-glioma interactions that contributed to glioma growth and invasion (121). Additionally, the MMP2 enzyme is released in a pro-form that needs to be cleaved to become active. The prominent enzyme for pro-MMP2 cleavage is the membrane-bound metalloprotease MT1- MMP. In this regard, slices obtained from MT1-MMP-deficient mice showed substantially smaller tumors. In addition, glioma growth was further reduced after microglia were removed from organotypic sections without MT1-MMP, suggesting that MT1-MMP is not the only glioma promoter expressed by microglia. In human glioma samples, MT1-MMP expression was positively correlated with the increasing malignancy of glioma (106).

### Wnt Signal Cascades

The Wingless-type MMTV integration site family (Wnt) proteins such as Wnt3a, Wnt5a, Wnt7a, Wnt5b, and Wnt2 participated in many biological processes (122). The Wnt signaling pathways consist of the β-catenin-independent pathway and the β-catenin-dependent pathway. The β-catenin-independent Wnt signaling pathway can be further divided into the Wnt/Planar Cell Polarity (PCP), Wnt/Calcium (Ca2+), and Wnt-dependent stabilization of proteins (STOP) signaling pathways (123). In the Wnt/β-catenin signaling pathway, Wnt proteins interact with the transmembrane receptor Frizzled and their co-receptor low density lipoprotein receptor-related protein 5/6 (LRP5/6), contributing to the stabilization of β catenin, its translocation to the nucleus, and consequent transcription of target genes essential to stem cell self-renewal, cell differentiation, polarization, and invasion 122,123. Increasing evidence indicated that Wnt signaling pathways play significant roles in the maintenance and progression of gliomas (124–126). Wnt signaling-induced proteins released from GSC mediate TAMs recruitment and M2-like polarization (70). In turn, Wnt proteins secreted from TAMs may further contribute to GBM stemness, mostly through the β-catenin dependent Wnt signaling, and may even increase its invasiveness and aggressiveness, mostly through β-catenin-independent Wnt signaling (127).

### Exosomes Signaling

Glioblastoma-derived exosomes (GDEs) can reprogram macrophages, converting M1 into TAMs and augmenting tumor-supportive functions of M2 macrophages. In turn, these GDEs-reprogrammed TAMs, release exosomes decorated by immunosuppressive and tumor-growth promoting proteins. TAM-derived exosomes disseminate these proteins in the TME contributing to tumor cell proliferation and migration. One study demonstrated that mechanisms underlying the promotion of glioblastoma growth involved Arginase-1+ exosomes produced by the reprogrammed TAMs. A selective Arginase-1 inhibitor, nor-NOHA reversed growth-promoting effects of arginase-1 carried by TAM-derived exosomes, suggesting that GBex-reprogrammed Arginase-1+ TAMs emerge as a major source of exosomes promoting tumor growth and as a potential therapeutic target in glioblastoma (128).

## TAMs Facilitate Angiogenesis of GBM

TAMs not only directly act on glioma cells, but also affect angiogenesis to indirectly impact tumor growth. In PTEN-null GBM models, TAMs secreted SSP1 (secreted phosphoprotein 1), which sustained glioma cell survival and stimulates angiogenesis (61). Signaling through the receptor for the advanced glycation end product (RAGE) was important for the process. RAGE ablation abrogated angiogenesis, which could be reconstituted with wild-type microglia or macrophages. Moreover, this TAMs activity correlated with the expression of VEGF, which is a critical pro-angiogenic factor (129). ADAM8, a metalloprotease-disintegrin strongly expressed in tumor cells and associated immune cells of GBMs is related to angiogenesis and is correlated with poor clinical prognosis. Furthermore, the angiogenic potential of ADAM8 in primary macrophages was mediated by the regulation of osteopontin (OPN), a crucial inducer of tumor angiogenesis. By *in vitro* cell signaling analyses, the study found that ADAM8 regulated OPN expression *via* JAK/STAT3 pathway in primary macrophages (130). M2-like immunosuppressive macrophages promote angiogenesis, whereas M1-like pro-inflammatory macrophages suppress angiogenesis. Zhu et al. showed that extracellular adenosine deaminase protein Cat Eye Syndrome Critical Region Protein 1 (CECR1) was highly expressed by M2-like macrophages in GBM where it defines macrophage M2 polarization and contributed to tumor expansion. Immunohistochemical evaluation of GBM tissue samples showed that the expression of CECR1 was correlated with microvascular density in the tumors. In a three-dimensional co-culture system consisting of human pericytes, human umbilical vein endothelial cells, and THP1-derived macrophages, CECR1 knockdown by siRNA and CECR1 stimulation of macrophages inhibited and promoted new vessel formation, respectively. Further investigation manifested that CECR1 function in (M2-like) macrophages mediated cross-talk between macrophages and pericytes in GBM *via* paracrine PDGFB–PDGFRβ signaling, promoting pericyte recruitment and migration, and tumor angiogenesis (131). In addition, Cui et al. observed that soluble macrophages-derived immunosuppressive cytokines, predominantly TGF-β1, and surface integrin (α_v_β_3_)-mediated endothelial macrophage interactions were required for inflammation-driven angiogenesis (132). The study demonstrated tuning cell-adhesion receptors using an integrin (α_v_β_3_)-specific collagen hydrogel regulated inflammation-driven angiogenesis through Src-PI3K-YAP signaling, highlighting the importance of altered cell-ECM interactions in inflammation. Dual integrin (α_v_β_3_) and cytokine receptor (TGFβ-R1) blockade suppressed GBM tumor neovascularization by simultaneously targeting macrophage-associated immunosuppression, endothelial-macrophage interactions, and altered ECM (132). Wang et al. validated that myeloid cell-restricted VEGF-A deficiency led to a growth delay of intracranial tumors and prolonged survival. Endothelial tube formation was significantly decreased by conditioned media from mutant macrophages (133). Recently, due to varied granulocyte influx, Blank et al. subdivided GBM samples into groups with low (GBM-lPMNL) and high numbers of granulocytes (GBM-hPMNL), which were related to activation of the microglia/macrophage population (134). Moreover, microglia/macrophages of the GBM-hPMNL specimens were highly associated with tumor blood vessels, accompanied by remodeling of the vascular structure. While microglia/macrophages represented the main source of alternative proangiogenic factors, additionally granulocytes participated through the production of IL8 and CD13, suggesting that tumor-infiltrating myeloid cells might play a crucial role for the limited efficacy of anti-angiogenic therapy bypassing VEGF mediated pathways through the expression of alternative proangiogenic factors (134).

## Potential Therapeutic Targets

For the past decades, investigators have largely focused on the intrinsic genetic mutations that occur in the tumor cells and the molecular mechanisms contributing to tumor progression. With the deepening understanding of TME in recent years, it is now acceptable that numerous signals emanated from the TME play pivotal roles in tumor growth. Concerning TME-mediated tumor aggression, it is important to recognize that glioblastoma is a sophisticated microcosm in which the interaction between neoplastic and non-neoplastic cells will not only affect gliomagenesis (135) but may also modify glioma responses to standard therapy. TAMs play critical roles in GBM growth and invasion, which provided a rationale for TAM-targeted therapies as feasible alternatives for GBM treatment. Generally, there are two strategies in terms of TAMs targeting therapies in GBM, including altering their pro-tumor function, often referred to as re-education, and blocking their recruitment.

### Re-Education of TAMs

BLZ945, a small-molecule CSF1R inhibitor, has been shown to ameliorate glioma progression by educating TAMs into an anti-tumor phenotype in a PN mouse model of GBM (136). However, further preclinical trials examining the long-term effect of BLZ945 reported rapid tumor rebound after a resting phase of 4 weeks (137). In detail, this resistance was mediated by TAMs *via* the excretion of insulin growth factor 1 (IGF-1) after the secretion of IL-4, probably produced by T cells in response to the drug. IGF-1 interacts with its cognate receptor IGF1R on the surface of tumor cells to activate the phosphatidylinositol 3-kinase (PI3K) signaling pathway, subsequently resulting in tumor resistance and proliferation (137). In a clinical trial with unselected adult recurrent GBM patients, unfortunately, CSF1R inhibitor as a single agent reported no effectiveness (138). A recent non-randomized, open-label, phase I/IIa, dose-escalation study targeting TAMs is at recruiting status, involving a single injection of Temferon, an investigational gene therapy-based approach consisting of autologous CD34+-enriched hematopoietic stem and progenitor cells exposed to transduction with a lentiviral vector driving myeloid-specific IFN-α2 expression (NCT03866109) (139). This strategy may provide a promising opportunity for GBM patients, as it showed prominent effectiveness in a mouse model of breast cancer.

### Blocking TAMs Recruitment

The CCL2/CCR2 signal pathway plays an essential role in monocyte recruitment toward the tumor niche. Downregulation of CCL2 levels prolonged the survival of GBM-bearing mice (40). Several clinical trials are currently underway to block CCL2 and CCR2 in solid tumors (140). Another promising target to block TAM recruitment is the SDF-1 receptor CXCR4. Some CXCR4 antagonists, such as peptide R or LY2510924, have demonstrated successful results in GBM mouse models (141, 142); However, they have not been validated in clinical trials. Another CXCR4 inhibitor, Plerixafor, has been tested for toxicity and efficacy in a completed Phase I/II clinical study in GBM patients after RT and temozolomide (NCT01977677). This study demonstrated that Plerixafor was well tolerated as adjunctive therapy for radiotherapy and chemotherapy in patients with newly-diagnosed GBM and improved the local control of tumor recurrence (143). Periostin has been reported as an interesting target for attenuating the tumor-supportive TAMs by interrupting integrin αvβ3 signaling (59). CD47 is another target to block TAM recruitment. Currently, there are two ongoing Phase I trials testing the efficacy of two monoclonal antibodies, IBI 188 (NCT03763149) and SRF-231 (NCT03512340), which are being conducted as monotherapies in patients with advanced malignant tumors and lymphomas.

## Conclusion

It is undeniable that there are complex and interdependent interactions exist between tumor cells and non-tumor cells within glioblastoma that promote the progression of GBM. As the main component of GBM, TAMs play an important role in the formation and growth of GBM. Although many emerging factors involved in TAMs and glioma cells interactions have been identified and tested in several pre-clinical studies over the past few years (**Table 1**), which factors are key to regulating this interesting interaction remains to be determined. Still, it is not clear how microglia and BMDMs interact in the tumor and whether they acquire different properties and perform different functions. It is not known whether histologically or molecularly different glioma types exhibit different functional phenotypes of TAMs. Nevertheless, targeting TAMs has emerged as a promising approach for GBM treatment. Further dissecting the mechanisms and interactions between TAMs and tumor cells or other immune cells will shed light on new GBM treatments. In addition, it is still needed to re-evaluate the efficacy of drugs that have been already used and investigated in the light of TAMs reprogramming. Integrating TAMs targeted therapies into available standard therapies or immunotherapies would be a promising field worthy of investigation.

**Table 1:** Interactions between Glioblastoma and GAMs.

Effects	Key factors	Mechanisms	References
**Glioblastoma on GAMs**			
**Recruitment**	CCL2	Chemokine	(40, 41, 45–47)
	CX3CL1	Chemokine	(49–53)
	CSF-1	Chemokine	(54, 55)
	HGF/SF	Chemokine	(56)
	CXCL12	Chemokine	(57)
	GDNF	Chemokine	(58)
	POSTN	GSCs secreted POSTN to recruit TAMs through the integrin αvβ₃	(59)
	OPN	OPN signals through the receptor Integrin αv β5 on TAMs	(60)
	LOX	LOX induced TAMs recruitment via activation of the b1 integrin-PYK2 pathway in macrophages	(61)
	CCN1	CCN1, a transcriptional target of YAP/TAZ, functions as a chemokine to recruit TAMs	(62)
	OLFML3	OLFML3 functions as a novel chemoattractant	(63)
	Kynurenine	Kynurenine activated aryl hydrocarbon receptor in TAMs, which promoted CCR2 expression, subsequently driving TAMs recruitment in response to CCL2	(64)
	EGFR/EGFRIII	EGFR and EGFRvIII cooperate to induce TAMs infiltration through KRAS-mediated upregulation of the chemokine CCL2	(65)
	ERK1/2	ERK1/2 mediate TAMS recruitment through regulation of the production of CCL2	(66)
	SETDB1	SETDB1 promoted AKT/mTOR-dependent CSF-1 induction and secretion, leading to macrophage recruitment in the tumor	(67)
	IL-33	IL-33 recruits TAMs through the regulation of chemokines	(68)
	RSK1	N/A	(69)
	WISP1	WISP1 signals through Integrin α6β1-Akt to recruit TAMs	(70)
**Pro-tumor Polarization**	S100B	S100B induced microglia activation through the induction of the STAT3 signal pathway	(77)
	PDAI3	PDIA3 induced microglia pro-tumor polarization toward the M2 phenotype and the secretion of pro-inflammatory factors	(78)
	ARS2	ARS2 activated its novel transcriptional target MGLL, encoding monoacylglycerol lipase (MAGL), stimulated M2-like TAM polarization through the production of PGE2	(79)
	CAIX	CAIX regulated through EGFR/STAT3/HIF-1α axis induced pro-tumor polarization of TAMs	(80)
	Romo1	Romo1 led to the M2 polarization of bone marrow-derived macrophages through the mTORC1 signaling pathway	(81)
	BCKAs	Exposure to BCKAs attenuated the phagocytic activity of macrophages	(82)
	mTOR	mTOR-mediated regulation of STAT3 and NF-κB activity promoted an immunosuppressive microglial phenotype	(83)
	IL-6	IL-6-activated STAT3 enhanced B7-H4 expression on TAMs, resulting in an immunosuppressive phenotype of TAMs	(84)
	GDEs	The components of GDEs such as IL-6 and miR-155-3p induced M2-like macrophage polarization through the IL-6-pSTAT3-miR-155-3p-autophagy-pSTAT3 positive feedback loop	(85–88)
	Versican	Versican-mediated TLR2 expression polarized microglia into a pro-tumorigenic phenotype featured by the upregulation of MT1-MMP and MMP9 expression	(120)
**GAMs on Glioblastoma**			
**Proliferation and invasion**	IL-6	IL-6 increased PDPK1-dependent PGK1 phosphorylation in glioblastoma cells, promoting tumor cell glycolysis and tumorigenesis	(107, 108)
	IL-1β	IL-1β activated phosphorylation of the glycolytic enzyme glycerol-3-phosphate dehydrogenase (GPD2) at threonine 10 (GPD2 pT10) through PI3K/PKCδ signal pathways to promote tumor growth	(109)
	STI1	N/A	(110)
	TGF-β2	TGF-β2 induced the expression of MMP2 and suppressed the expression of (TIMP)-2 to promote glioma invasion	(111, 112)
	CECR1	CECR1 stimulated MAPK signaling and activated the proliferation and migration of glioma cells	(113)
	PTN	PTN promoted GBM malignant growth through PTN–PTPRZ1 paracrine signaling	(114)
	CCL4	CCL4-CCR5 axis participated in TAMs-mediated glioblastoma invasion	(115)
	CCL5	CCL5 upregulated mmp2 through the CaMKII and p-Akt signals	(116)
	CCL8	CCL8 dramatically activated ERK1/2 phosphorylation in GBM cells and promoted invasion and stemlike traits of GBM cells through CCR1 and CCR5	(117)
	TLR2	TLR2 forms heterodimers with TLR1 and TLR6 modulating MT1-MMP expression to promote tumor invasion and growth	(119)
	Wnt	TAMs secreted Wnt proteins, contributing to GBM invasiveness and aggressiveness mostly through β-catenin-independent Wnt signaling	(124–126)
	Exosomes	GBex-reprogrammed Arginase-1+ TAMs emerge as a major source of exosomes promoting tumor growth	(128)
**Angiogenesis**	SSP1	N/A	(61)
	RAGE	N/A	(129)
	ADAM8	ADAM8 induced angiogenesis via JAK/STAT3 pathway mediated OPN expression	(130)
	CECR1	CECR1 promoted pericyte recruitment and migration, and tumor angiogenesis via paracrine PDGFB–PDGFRβ signaling,	(131)
	TGF-β1	TGF-β1/integrin (α_v_β_3_) interaction between macrophages and endothelial promoted GBM angiogenesis	(132)
	VEGF-A	N/A	(133)

## Author Contributions

All authors listed have made a substantial, direct, and intellectual contribution to the work and approved it for publication.

## Conflict of Interest

The authors declare that the research was conducted in the absence of any commercial or financial relationships that could be construed as a potential conflict of interest.

## Publisher’s Note

All claims expressed in this article are solely those of the authors and do not necessarily represent those of their affiliated organizations, or those of the publisher, the editors and the reviewers. Any product that may be evaluated in this article, or claim that may be made by its manufacturer, is not guaranteed or endorsed by the publisher.

## Glossary

**Table:**

ADAM8	a disintegrin and metalloprotease
AHR	aryl hydrocarbon receptor
ARS2	arsenite-resistance protein 2
BCKAs	branched-chain ketoacids
BMDM	bone marrow-derived macrophages
CAIX	carbonic anhydrase IX
CCL	chemokine (C-C motif) ligand
CCN1	cellular communication network factor 1
CECR1	Cat Eye Syndrome Critical Region Protein 1
CSF-1	colony stimulating factor-1
DGCs	differentiated Glioblastoma Cells
EGFR	epidermal growth factor receptor
FAS	fatty acid synthesis
GAMs	glioblastoma-associated macrophages
GBM	glioblastoma multiform
GDEs	glioblastoma-derived exosomes
GDNF	growth factor glial cell-derived neurotrophic factor
GM-CSF	granulocyte-macrophage colony-stimulating factor
GPD2 pT10	glycolytic enzyme glycerol-3-phosphate dehydrogenase (GPD2) at threonine 10
GPD2	glycerol-3-phosphate dehydrogenase 2
GSCs	glioblastoma stem cells
H-GDEs	hypoxic glioma-derived exosomes
HGF	hepatocyte growth factor
IGF-1	insulin growth factor 1
IL-1β	interleukin 1β
IL-6	interleukin 6
IRF-8	interferon regulatory factor-8
ITGαvβ5	Integrin αv β5
LOX	lysyl oxidase
LPS	lipopolysaccharide
LRP	lipoprotein receptor-related protein
MAGL	monoacylglycerol lipase
MCP-1	monocyte chemoattractant protein-1
MMP2	matrix metalloprotease-2
N-GDEs	normoxic glioma-derived exosomes
OPN	osteopontin
PD-L1	programmed death-ligand 1
PGE2	prostaglandin E2
PI3K	phosphatidylinositol 3-kinase
PKCδ	protein kinase-delta
POSTN	periostin
PTN	pleiotrophin
PCP	Planar Cell Polarity
RAGE	advanced glycation end product
Romo1	reactive oxygen species modulator 1
RSK1	ribosomal S6 kinase 1
SETDB1	SET domain bifurcated 1
SF	scatter factor
SSP1	secreted phosphoprotein 1
STAT3	signal transducer and activator of transcription 3
STI1	stress-inducible protein 1
TAMs	tumor-associated macrophages
TGF-β	Transforming growth factor-β
TIMP	tissue inhibitors of metalloproteinases
TME	tumor microenvironment
WISP1	Wnt‐induced signaling protein 1
Wnt	The Wingless-type MMTV integration site family

## References

[1] Ostrom QT Gittleman H de Blank PM Finlay JL Gurney JG McKean-Cowdin R. American Brain Tumor Association Adolescent and Young Adult Primary Brain and Central Nervous System Tumors Diagnosed in the United States in 2008-2012. Neuro Oncol (2016) 18 Suppl 1:i1–i50. doi: 10.1093/neuonc/nov297 26705298 PMC4690545

[2] Ostrom QT Gittleman H Fulop J Liu M Blanda R Kromer C. CBTRUS Statistical Report: Primary Brain and Central Nervous System Tumors Diagnosed in the United States in 2008-2012. Neuro Oncol (2015) 17 Suppl 4:iv1–62. doi: 10.1093/neuonc/nov189 26511214 PMC4623240

[3] Geraldo LHM Garcia C da Fonseca ACC Dubois LGF de Sampaio ESTCL Matias D. Glioblastoma Therapy in the Age of Molecular Medicine. Trends Cancer (2019) 5(1):46–65. doi: 10.1016/j.trecan.2018.11.002 30616755

[4] Bejarano L Jordao MJC Joyce JA. Therapeutic Targeting of the Tumor Microenvironment. Cancer Discov (2021) 11(4):933–59. doi: 10.1158/2159-8290.CD-20-1808 33811125

[5] Buonfiglioli A Hambardzumyan D. Macrophages and Microglia: The Cerberus of Glioblastoma. Acta Neuropathol Commun (2021) 9(1):54. doi: 10.1186/s40478-021-01156-z 33766119 PMC7992800

[6] Morisse MC Jouannet S Dominguez-Villar M Sanson M Idbaih A. Interactions Between Tumor-Associated Macrophages and Tumor Cells in Glioblastoma: Unraveling Promising Targeted Therapies. Expert Rev Neurother (2018) 18(9):729–37. doi: 10.1080/14737175.2018.1510321 30099909

[7] Andersen RS Anand A Harwood DSL Kristensen BW. Tumor-Associated Microglia and Macrophages in the Glioblastoma Microenvironment and Their Implications for Therapy. Cancers (Basel) (2021) 13(17):4255. doi: 10.3390/cancers13174255 34503065 PMC8428223

[8] Wei J Chen P Gupta P Ott M Zamler D Kassab C. Immune Biology of Glioma-Associated Macrophages and Microglia: Functional and Therapeutic Implications. Neuro Oncol (2020) 22(2):180–94. doi: 10.1093/neuonc/noz212 PMC7442334 31679017

[9] Mosser DM Edwards JP. Exploring the Full Spectrum of Macrophage Activation. Nat Rev Immunol (2008) 8(12):958–69. doi: 10.1038/nri2448 PMC2724991 19029990

[10] Locati M Curtale G Mantovani A. Diversity, Mechanisms, and Significance of Macrophage Plasticity. Annu Rev Pathol (2020) 15:123–47. doi: 10.1146/annurev-pathmechdis-012418-012718 PMC7176483 31530089

[11] Hambardzumyan D Gutmann DH Kettenmann H. The Role of Microglia and Macrophages in Glioma Maintenance and Progression. Nat Neurosci (2016) 19(1):20–7. doi: 10.1038/nn.4185 PMC4876023 26713745

[12] Gutmann DH Kettenmann H. Microglia/Brain Macrophages as Central Drivers of Brain Tumor Pathobiology. Neuron (2019) 104(3):442–9. doi: 10.1016/j.neuron.2019.08.028 PMC7288606 31697921

[13] Ginhoux F Greter M Leboeuf M Nandi S See P Gokhan S. Fate Mapping Analysis Reveals That Adult Microglia Derive From Primitive Macrophages. Science (2010) 330(6005):841–5. doi: 10.1126/science.1194637 PMC3719181 20966214

[14] De S Van Deren D Peden E Hockin M Boulet A Titen S. Two Distinct Ontogenies Confer Heterogeneity to Mouse Brain Microglia. Development (2018) 145(13):dev152306. doi: 10.1242/dev.152306 29973370 PMC6053660

[15] Herisson F Frodermann V Courties G Rohde D Sun Y Vandoorne K. Direct Vascular Channels Connect Skull Bone Marrow and the Brain Surface Enabling Myeloid Cell Migration. Nat Neurosci (2018) 21(9):1209–17. doi: 10.1038/s41593-018-0213-2 PMC6148759 30150661

[16] Ling EA Wong WC. The Origin and Nature of Ramified and Amoeboid Microglia: A Historical Review and Current Concepts. Glia (1993) 7(1):9–18. doi: 10.1002/glia.440070105 8423067

[17] Brandenburg S Blank A Bungert AD Vajkoczy P. Distinction of Microglia and Macrophages in Glioblastoma: Close Relatives, Different Tasks? Int J Mol Sci (2020) 22(1):194. doi: 10.3390/ijms22010194 PMC7794706 33375505

[18] Biffi A De Palma M Quattrini A Del Carro U Amadio S Visigalli I. Correction of Metachromatic Leukodystrophy in the Mouse Model by Transplantation of Genetically Modified Hematopoietic Stem Cells. J Clin Invest (2004) 113(8):1118–29. doi: 10.1172/JCI200419205 PMC385395 15085191

[19] Simard AR Rivest S. Bone Marrow Stem Cells Have the Ability to Populate the Entire Central Nervous System Into Fully Differentiated Parenchymal Microglia. FASEB J (2004) 18(9):998–1000. doi: 10.1096/fj.04-1517fje 15084516

[20] Priller J Flugel A Wehner T Boentert M Haas CA Prinz M. Targeting Gene-Modified Hematopoietic Cells to the Central Nervous System: Use of Green Fluorescent Protein Uncovers Microglial Engraftment. Nat Med (2001) 7(12):1356–61. doi: 10.1038/nm1201-1356 11726978

[21] Hickey WF Kimura H. Perivascular Microglial Cells of the CNS Are Bone Marrow-Derived and Present Antigen In Vivo. Science (1988) 239(4837):290–2. doi: 10.1126/science.3276004 3276004

[22] Flugel A Bradl M Kreutzberg GW Graeber MB. Transformation of Donor-Derived Bone Marrow Precursors Into Host Microglia During Autoimmune CNS Inflammation and During the Retrograde Response to Axotomy. J Neurosci Res (2001) 66(1):74–82. doi: 10.1002/jnr.1198 11599003

[23] Massengale M Wagers AJ Vogel H Weissman IL. Hematopoietic Cells Maintain Hematopoietic Fates Upon Entering the Brain. J Exp Med (2005) 201(10):1579–89. doi: 10.1084/jem.20050030 PMC2212913 15897275

[24] Ajami B Bennett JL Krieger C Tetzlaff W Rossi FM. Local Self-Renewal can Sustain CNS Microglia Maintenance and Function Throughout Adult Life. Nat Neurosci (2007) 10(12):1538–43. doi: 10.1038/nn2014 18026097

[25] Ajami B Bennett JL Krieger C McNagny KM Rossi FM. Infiltrating Monocytes Trigger EAE Progression, But do Not Contribute to the Resident Microglia Pool. Nat Neurosci (2011) 14(9):1142–9. doi: 10.1038/nn.2887 21804537

[26] Elmore MR Najafi AR Koike MA Dagher NN Spangenberg EE Rice RA. Colony-Stimulating Factor 1 Receptor Signaling Is Necessary for Microglia Viability, Unmasking a Microglia Progenitor Cell in the Adult Brain. Neuron (2014) 82(2):380–97. doi: 10.1016/j.neuron.2014.02.040 PMC4161285 24742461

[27] Ochocka N Segit P Walentynowicz KA Wojnicki K Cyranowski S Swatler J. Single-Cell RNA Sequencing Reveals Functional Heterogeneity of Glioma-Associated Brain Macrophages. Nat Commun (2021) 12(1):1151. doi: 10.1038/s41467-021-21407-w 33608526 PMC7895824

[28] Grabert K Michoel T Karavolos MH Clohisey S Baillie JK Stevens MP. Microglial Brain Region-Dependent Diversity and Selective Regional Sensitivities to Aging. Nat Neurosci (2016) 19(3):504–16. doi: 10.1038/nn.4222 PMC4768346 26780511

[29] Yona S Kim KW Wolf Y Mildner A Varol D Breker M. Fate Mapping Reveals Origins and Dynamics of Monocytes and Tissue Macrophages Under Homeostasis. Immunity (2013) 38(1):79–91. doi: 10.1016/j.immuni.2012.12.001 23273845 PMC3908543

[30] Hoeffel G Chen J Lavin Y Low D Almeida FF See P. C-Myb(+) Erythro-Myeloid Progenitor-Derived Fetal Monocytes Give Rise to Adult Tissue-Resident Macrophages. Immunity (2015) 42(4):665–78. doi: 10.1016/j.immuni.2015.03.011 PMC4545768 25902481

[31] Hoeffel G Ginhoux F. Fetal Monocytes and the Origins of Tissue-Resident Macrophages. Cell Immunol (2018) 330:5–15. doi: 10.1016/j.cellimm.2018.01.001 29475558

[32] Geissmann F Jung S Littman DR. Blood Monocytes Consist of Two Principal Subsets With Distinct Migratory Properties. Immunity. (2003) 19(1):71–82. doi: 10.1016/S1074-7613(03)00174-2 12871640

[33] Varol C Mildner A Jung S. Macrophages: Development and Tissue Specialization. Annu Rev Immunol (2015) 33:643–75. doi: 10.1146/annurev-immunol-032414-112220 25861979

[34] Swirski FK Nahrendorf M Etzrodt M Wildgruber M Cortez-Retamozo V Panizzi P. Identification of Splenic Reservoir Monocytes and Their Deployment to Inflammatory Sites. Science. (2009) 325(5940):612–6. doi: 10.1126/science.1175202 PMC2803111 19644120

[35] Ginhoux F Guilliams M. Tissue-Resident Macrophage Ontogeny and Homeostasis. Immunity (2016) 44(3):439–49. doi: 10.1016/j.immuni.2016.02.024 26982352

[36] Ginhoux F Jung S. Monocytes and Macrophages: Developmental Pathways and Tissue Homeostasis Nat Rev Immunol (2014) 14(6):392–404. doi: 10.1038/nri3671 24854589

[37] Davies LC Jenkins SJ Allen JE Taylor PR. Tissue-Resident Macrophages. Nat Immunol (2013) 14(10):986–95. doi: 10.1038/ni.2705 PMC4045180 24048120

[38] Shi C Pamer EG. Monocyte Recruitment During Infection and Inflammation. Nat Rev Immunol (2011) 11(11):762–74. doi: 10.1038/nri3070 PMC3947780 21984070

[39] Wolf SA Boddeke HW Kettenmann H. Microglia in Physiology and Disease. Annu Rev Physiol (2017) 79:619–43. doi: 10.1146/annurev-physiol-022516-034406 27959620

[40] Chen Z Feng X Herting CJ Garcia VA Nie K Pong WW. Cellular and Molecular Identity of Tumor-Associated Macrophages in Glioblastoma. Cancer Res (2017) 77(9):2266–78. doi: 10.1158/0008-5472.CAN-16-2310 PMC5741820 28235764

[41] Hutter G Theruvath J Graef CM Zhang M Schoen MK Manz EM. Microglia Are Effector Cells of CD47-SIRPalpha Antiphagocytic Axis Disruption Against Glioblastoma. Proc Natl Acad Sci USA (2019) 116(3):997–1006. doi: 10.1073/pnas.1721434116 30602457 PMC6338872

[42] Yu K Youshani AS Wilkinson FL O'Leary C Cook P Laaniste L. A Nonmyeloablative Chimeric Mouse Model Accurately Defines Microglia and Macrophage Contribution in Glioma. Neuropathol Appl Neurobiol (2019) 45(2):119–40. doi: 10.1111/nan.12489 PMC7379954 29679380

[43] Nagarsheth N Wicha MS Zou W. Chemokines in the Cancer Microenvironment and Their Relevance in Cancer Immunotherapy. Nat Rev Immunol (2017) 17(9):559–72. doi: 10.1038/nri.2017.49 PMC5731833 28555670

[44] Vakilian A Khorramdelazad H Heidari P Sheikh Rezaei Z Hassanshahi G. CCL2/CCR2 Signaling Pathway in Glioblastoma Multiforme. Neurochem Int (2017) 103:1–7. doi: 10.1016/j.neuint.2016.12.013 28025034

[45] Platten M Kretz A Naumann U Aulwurm S Egashira K Isenmann S. Monocyte Chemoattractant Protein-1 Increases Microglial Infiltration and Aggressiveness of Gliomas. Ann Neurol (2003) 54(3):388–92. doi: 10.1002/ana.10679 12953273

[46] Felsenstein M Blank A Bungert AD Mueller A Ghori A Kremenetskaia I. CCR2 of Tumor Microenvironmental Cells Is a Relevant Modulator of Glioma Biology. Cancers (Basel) (2020) 12(7):1882. doi: 10.3390/cancers12071882 PMC7408933 32668709

[47] Jung Y Ahn SH Park H Park SH Choi K Choi C. MCP-1 and MIP-3alpha Secreted From Necrotic Cell-Treated Glioblastoma Cells Promote Migration/Infiltration of Microglia. Cell Physiol Biochem (2018) 48(3):1332–46. doi: 10.1159/000492092 30048972

[48] Okada M Saio M Kito Y Ohe N Yano H Yoshimura S. Tumor-Associated Macrophage/Microglia Infiltration in Human Gliomas Is Correlated With MCP-3, But Not MCP-1. Int J Oncol (2009) 34(6):1621–7. doi: 10.3892/ijo_00000292 19424580

[49] Paolicelli RC Bisht K Tremblay ME. Fractalkine Regulation of Microglial Physiology and Consequences on the Brain and Behavior. Front Cell Neurosci (2014) 8:129. doi: 10.3389/fncel.2014.00129 24860431 PMC4026677

[50] Feng X Szulzewsky F Yerevanian A Chen Z Heinzmann D Rasmussen RD. Loss of CX3CR1 Increases Accumulation of Inflammatory Monocytes and Promotes Gliomagenesis. Oncotarget (2015) 6(17):15077–94. doi: 10.18632/oncotarget.3730 PMC4558137 25987130

[51] Liu C Luo D Streit WJ Harrison JK. CX3CL1 and CX3CR1 in the GL261 Murine Model of Glioma: CX3CR1 Deficiency Does Not Impact Tumor Growth or Infiltration of Microglia and Lymphocytes. J Neuroimmunol (2008) 198(1-2):98–105. doi: 10.1016/j.jneuroim.2008.04.016 18508133 PMC2561213

[52] Rodero M Marie Y Coudert M Blondet E Mokhtari K Rousseau A. Polymorphism in the Microglial Cell-Mobilizing CX3CR1 Gene Is Associated With Survival in Patients With Glioblastoma. J Clin Oncol (2008) 26(36):5957–64. doi: 10.1200/JCO.2008.17.2833 19001328

[53] Guo X Pan Y Gutmann DH. Genetic and Genomic Alterations Differentially Dictate Low-Grade Glioma Growth Through Cancer Stem Cell-Specific Chemokine Recruitment of T Cells and Microglia. Neuro Oncol (2019) 21(10):1250–62. doi: 10.1093/neuonc/noz080 PMC6784288 31111915

[54] Coniglio SJ Eugenin E Dobrenis K Stanley ER West BL Symons MH. Microglial Stimulation of Glioblastoma Invasion Involves Epidermal Growth Factor Receptor (EGFR) and Colony Stimulating Factor 1 Receptor (CSF-1R) Signaling. Mol Med (2012) 18:519–27. doi: 10.2119/molmed.2011.00217 PMC3356419 22294205

[55] Sielska M Przanowski P Wylot B Gabrusiewicz K Maleszewska M Kijewska M. Distinct Roles of CSF Family Cytokines in Macrophage Infiltration and Activation in Glioma Progression and Injury Response. J Pathol (2013) 230(3):310–21. doi: 10.1002/path.4192 23520016

[56] Badie B Schartner J Klaver J Vorpahl J. *In Vitro* Modulation of Microglia Motility by Glioma Cells Is Mediated by Hepatocyte Growth Factor/Scatter Factor. Neurosurgery (1999) 44(5):1077–82;. discussion doi: 10.1097/00006123-199905000-00075 10232541

[57] Wang SC Hong JH Hsueh C Chiang CS. Tumor-Secreted SDF-1 Promotes Glioma Invasiveness and TAM Tropism Toward Hypoxia in a Murine Astrocytoma Model. Lab Invest (2012) 92(1):151–62. doi: 10.1038/labinvest.2011.128 21894147

[58] Ku MC Wolf SA Respondek D Matyash V Pohlmann A Waiczies S. GDNF Mediates Glioblastoma-Induced Microglia Attraction But Not Astrogliosis. Acta Neuropathol (2013) 125(4):609–20. doi: 10.1007/s00401-013-1079-8 23344256

[59] Zhou W Ke SQ Huang Z Flavahan W Fang X Paul J. Periostin Secreted by Glioblastoma Stem Cells Recruits M2 Tumour-Associated Macrophages and Promotes Malignant Growth. Nat Cell Biol (2015) 17(2):170–82. doi: 10.1038/ncb3090 PMC4312504 25580734

[60] Wei J Marisetty A Schrand B Gabrusiewicz K Hashimoto Y Ott M. Osteopontin Mediates Glioblastoma-Associated Macrophage Infiltration and Is a Potential Therapeutic Target. J Clin Invest (2019) 129(1):137–49. doi: 10.1172/JCI121266 PMC6307970 30307407

[61] Chen P Zhao D Li J Liang X Li J Chang A. Symbiotic Macrophage-Glioma Cell Interactions Reveal Synthetic Lethality in PTEN-Null Glioma. Cancer Cell (2019) 35(6):868–84.e6. doi: 10.1016/j.ccell.2019.05.003 31185211 PMC6561349

[62] Uneda A Kurozumi K Fujimura A Fujii K Ishida J Shimazu Y. Differentiated Glioblastoma Cells Accelerate Tumor Progression by Shaping the Tumor Microenvironment *via* CCN1-Mediated Macrophage Infiltration. Acta Neuropathol Commun (2021) 9(1):29. doi: 10.1186/s40478-021-01124-7 33618763 PMC7898455

[63] Chen P Hsu WH Chang A Tan Z Lan Z Zhou A. Circadian Regulator CLOCK Recruits Immune-Suppressive Microglia Into the GBM Tumor Microenvironment. Cancer Discov (2020) 10(3):371–81. doi: 10.1158/2159-8290.CD-19-0400 PMC7058515 31919052

[64] Takenaka MC Gabriely G Rothhammer V Mascanfroni ID Wheeler MA Chao CC. Control of Tumor-Associated Macrophages and T Cells in Glioblastoma *via* AHR and CD39. Nat Neurosci (2019) 22(5):729–40. doi: 10.1038/s41593-019-0370-y PMC8052632 30962630

[65] An Z Knobbe-Thomsen CB Wan X Fan QW Reifenberger G Weiss WA. EGFR Cooperates With EGFRvIII to Recruit Macrophages in Glioblastoma. Cancer Res (2018) 78(24):6785–94. doi: 10.1158/0008-5472.CAN-17-3551 PMC6295222 30401716

[66] Lailler C Louandre C Morisse MC Lhossein T Godin C Lottin M. ERK1/2 Signaling Regulates the Immune Microenvironment and Macrophage Recruitment in Glioblastoma. Biosci Rep (2019) 39(9):BSR20191433. doi: 10.1042/BSR20191433 31467175 PMC6744584

[67] Han S Zhen W Guo T Zou J Li F. SETDB1 Promotes Glioblastoma Growth *via* CSF-1-Dependent Macrophage Recruitment by Activating the AKT/mTOR Signaling Pathway. J Exp Clin Cancer Res (2020) 39(1):218. doi: 10.1186/s13046-020-01730-8 33059737 PMC7560339

[68] De Boeck A Ahn BY D'Mello C Lun X Menon SV Alshehri MM. Glioma-Derived IL-33 Orchestrates an Inflammatory Brain Tumor Microenvironment That Accelerates Glioma Progression. Nat Commun (2020) 11(1):4997. doi: 10.1038/s41467-020-18569-4 33020472 PMC7536425

[69] Hajj GNM da Silva FF de Bellis B Lupinacci FCS Bellato HM Cruz JR. Aberrant Expression of RSK1 Characterizes High-Grade Gliomas With Immune Infiltration. Mol Oncol (2020) 14(1):159–79. doi: 10.1002/1878-0261.12595 PMC6944115 31701625

[70] Tao W Chu C Zhou W Huang Z Zhai K Fang X. Dual Role of WISP1 in Maintaining Glioma Stem Cells and Tumor-Supportive Macrophages in Glioblastoma. Nat Commun (2020) 11(1):3015. doi: 10.1038/s41467-020-16827-z 32541784 PMC7295765

[71] Gordon S Martinez FO. Alternative Activation of Macrophages: Mechanism and Functions. Immunity (2010) 32(5):593–604. doi: 10.1016/j.immuni.2010.05.007 20510870

[72] Shapouri-Moghaddam A Mohammadian S Vazini H Taghadosi M Esmaeili SA Mardani F. Macrophage Plasticity, Polarization, and Function in Health and Disease. J Cell Physiol (2018) 233(9):6425–40. doi: 10.1002/jcp.26429 PMC13482560 29319160

[73] Mantovani A Sozzani S Locati M Allavena P Sica A. Macrophage Polarization: Tumor-Associated Macrophages as a Paradigm for Polarized M2 Mononuclear Phagocytes. Trends Immunol (2002) 23(11):549–55. doi: 10.1016/S1471-4906(02)02302-5 12401408

[74] Chen Z Herting CJ Ross JL Gabanic B Puigdelloses Vallcorba M Szulzewsky F. Genetic Driver Mutations Introduced in Identical Cell-of-Origin in Murine Glioblastoma Reveal Distinct Immune Landscapes But Similar Response to Checkpoint Blockade. Glia (2020) 68(10):2148–66. doi: 10.1002/glia.23883 PMC7512141 32639068

[75] Szulzewsky F Pelz A Feng X Synowitz M Markovic D Langmann T. Glioma-Associated Microglia/Macrophages Display an Expression Profile Different From M1 and M2 Polarization and Highly Express Gpnmb and Spp1. PloS One (2015) 10(2):e0116644. doi: 10.1371/journal.pone.0116644 25658639 PMC4320099

[76] Gabrusiewicz K Rodriguez B Wei J Hashimoto Y Healy LM Maiti SN. Glioblastoma-Infiltrated Innate Immune Cells Resemble M0 Macrophage Phenotype. JCI Insight (2016) 1(2):e85841. doi: 10.1172/jci.insight.85841 PMC4784261 26973881

[77] Gao H Zhang IY Zhang L Song Y Liu S Ren H. S100B Suppression Alters Polarization of Infiltrating Myeloid-Derived Cells in Gliomas and Inhibits Tumor Growth. Cancer Lett (2018) 439:91–100. doi: 10.1016/j.canlet.2018.07.034 30076898 PMC7048242

[78] Chiavari M Ciotti GMP Canonico F Altieri F Lacal PM Graziani G. PDIA3 Expression in Glioblastoma Modulates Macrophage/Microglia Pro-Tumor Activation. Int J Mol Sci (2020) 21(21):8214. doi: 10.3390/ijms21218214 PMC7662700 33153019

[79] Yin J Kim SS Choi E Oh YT Lin W Kim TH. ARS2/MAGL Signaling in Glioblastoma Stem Cells Promotes Self-Renewal and M2-Like Polarization of Tumor-Associated Macrophages. Nat Commun (2020) 11(1):2978. doi: 10.1038/s41467-020-16789-2 32532977 PMC7293269

[80] Huang BR Liu YS Lai SW Lin HJ Shen CK Yang LY. CAIX Regulates GBM Motility and TAM Adhesion and Polarization Through EGFR/STAT3 Under Hypoxic Conditions. Int J Mol Sci (2020) 21(16):5838. doi: 10.3390/ijms21165838 PMC7461579 32823915

[81] Sun G Cao Y Qian C Wan Z Zhu J Guo J. Romo1 Is Involved in the Immune Response of Glioblastoma by Regulating the Function of Macrophages. Aging (Albany NY) (2020) 12(2):1114–27. doi: 10.18632/aging.102648 PMC7053633 31945745

[82] Silva LS Poschet G Nonnenmacher Y Becker HM Sapcariu S Gaupel AC. Branched-Chain Ketoacids Secreted by Glioblastoma Cells *via* MCT1 Modulate Macrophage Phenotype. EMBO Rep (2017) 18(12):2172–85. doi: 10.15252/embr.201744154 PMC5709768 29066459

[83] Dumas AA Pomella N Rosser G Guglielmi L Vinel C Millner TO. Microglia Promote Glioblastoma *via* mTOR-Mediated Immunosuppression of the Tumour Microenvironment. EMBO J (2020) 39(15):e103790. doi: 10.15252/embj.2019103790 32567735 PMC7396846

[84] Yao Y Ye H Qi Z Mo L Yue Q Baral A. B7-H4(B7x)-Mediated Cross-Talk Between Glioma-Initiating Cells and Macrophages *via* the IL6/JAK/STAT3 Pathway Lead to Poor Prognosis in Glioma Patients. Clin Cancer Res (2016) 22(11):2778–90. doi: 10.1158/1078-0432.CCR-15-0858 PMC4891287 27001312

[85] Moradi-Chaleshtori M Hashemi SM Soudi S Bandehpour M Mohammadi-Yeganeh S. Tumor-Derived Exosomal microRNAs and Proteins as Modulators of Macrophage Function. J Cell Physiol (2019) 234(6):7970–82. doi: 10.1002/jcp.27552 30378104

[86] Baig MS Roy A Rajpoot S Liu D Savai R Banerjee S. Tumor-Derived Exosomes in the Regulation of Macrophage Polarization. Inflamm Res (2020) 69(5):435–51. doi: 10.1007/s00011-020-01318-0 32162012

[87] Xu J Zhang J Zhang Z Gao Z Qi Y Qiu W. Hypoxic Glioma-Derived Exosomes Promote M2-Like Macrophage Polarization by Enhancing Autophagy Induction. Cell Death Dis (2021) 12(4):373. doi: 10.1038/s41419-021-03664-1 33828078 PMC8026615

[88] Gabrusiewicz K Li X Wei J Hashimoto Y Marisetty AL Ott M. Glioblastoma Stem Cell-Derived Exosomes Induce M2 Macrophages and PD-L1 Expression on Human Monocytes. Oncoimmunology (2018) 7(4):e1412909. doi: 10.1080/2162402X.2017.1412909 29632728 PMC5889290

[89] Maleszewska M Steranka A Smiech M Kaza B Pilanc P Dabrowski M. Sequential Changes in Histone Modifications Shape Transcriptional Responses Underlying Microglia Polarization by Glioma. Glia (2021) 69(1):109–23. doi: 10.1002/glia.23887 32710676

[90] Larionova I Cherdyntseva N Liu T Patysheva M Rakina M Kzhyshkowska J. Interaction of Tumor-Associated Macrophages and Cancer Chemotherapy. Oncoimmunology (2019) 8(7):1596004. doi: 10.1080/2162402X.2019.1596004 31143517 PMC6527283

[91] Gregoire H Roncali L Rousseau A Cherel M Delneste Y Jeannin P. Targeting Tumor Associated Macrophages to Overcome Conventional Treatment Resistance in Glioblastoma. Front Pharmacol (2020) 11:368. doi: 10.3389/fphar.2020.00368 32322199 PMC7158850

[92] Gupta K. Chapter 13 - The Molecular and Cellular Effects of Radiotherapy-Induced Microenvironment Changes on Potential Chemoresistance in Glioblastoma. In: Paulmurugan R Massoud TF, editors. Glioblastoma Resistance to Chemotherapy: Molecular Mechanisms and Innovative Reversal Strategies, *vol*. 15. Academic Press (2021). p. 335–64.

[93] Tabatabaei P Visse E Bergstrom P Brannstrom T Siesjo P Bergenheim AT. Radiotherapy Induces an Immediate Inflammatory Reaction in Malignant Glioma: A Clinical Microdialysis Study. J Neurooncol (2017) 131(1):83–92. doi: 10.1007/s11060-016-2271-1 27664151 PMC5258803

[94] De Palma M Lewis CE. Cancer: Macrophages Limit Chemotherapy. Nature (2011) 472(7343):303–4. doi: 10.1038/472303a 21512566

[95] Zheng X Mansouri S Krager A Grimminger F Seeger W Pullamsetti SS. Metabolism in Tumour-Associated Macrophages: A Quid Pro Quo With the Tumour Microenvironment. Eur Respir Rev (2020) 29(157):200134. doi: 10.1183/16000617.0134-2020 33004525 PMC9488699

[96] Mazzone M Menga A Castegna A. Metabolism and TAM Functions-It Takes Two to Tango. FEBS J (2018) 285(4):700–16. doi: 10.1111/febs.14295 29055087

[97] Puthenveetil A Dubey S. Metabolic Reprograming of Tumor-Associated Macrophages. Ann Transl Med (2020) 8(16):1030. doi: 10.21037/atm-20-2037 32953830 PMC7475460

[98] Qian BZ Pollard JW. Macrophage Diversity Enhances Tumor Progression and Metastasis. Cell (2010) 141(1):39–51. doi: 10.1016/j.cell.2010.03.014 20371344 PMC4994190

[99] Condeelis J Pollard JW. Macrophages: Obligate Partners for Tumor Cell Migration, Invasion, and Metastasis. Cell (2006) 124(2):263–6. doi: 10.1016/j.cell.2006.01.007 16439202

[100] Kashfi K Kannikal J Nath N. Macrophage Reprogramming and Cancer Therapeutics: Role of iNOS-Derived No. Cells (2021) 10(11):3194. doi: 10.3390/cells10113194 34831416 PMC8624911

[101] Won WJ Deshane JS Leavenworth JW Oliva CR Griguer CE. Metabolic and Functional Reprogramming of Myeloid-Derived Suppressor Cells and Their Therapeutic Control in Glioblastoma. Cell Stress (2019) 3(2):47–65. doi: 10.15698/cst2019.02.176 31225500 PMC6551710

[102] Carroll RG Zaslona Z Galvan-Pena S Koppe EL Sevin DC Angiari S. An Unexpected Link Between Fatty Acid Synthase and Cholesterol Synthesis in Proinflammatory Macrophage Activation. J Biol Chem (2018) 293(15):5509–21. doi: 10.1074/jbc.RA118.001921 PMC5900750 29463677

[103] Baik SH Kang S Lee W Choi H Chung S Kim JI. A Breakdown in Metabolic Reprogramming Causes Microglia Dysfunction in Alzheimer's Disease. Cell Metab (2019) 30(3):493–507.e6. doi: 10.1016/j.cmet.2019.06.005 31257151

[104] Bettinger I Thanos S Paulus W. Microglia Promote Glioma Migration. Acta Neuropathol (2002) 103(4):351–5. doi: 10.1007/s00401-001-0472-x 11904754

[105] Markovic DS Glass R Synowitz M Rooijen N Kettenmann H. Microglia Stimulate the Invasiveness of Glioma Cells by Increasing the Activity of Metalloprotease-2. J Neuropathol Exp Neurol (2005) 64(9):754–62. doi: 10.1097/01.jnen.0000178445.33972.a9 16141784

[106] Markovic DS Vinnakota K Chirasani S Synowitz M Raguet H Stock K. Gliomas Induce and Exploit Microglial MT1-MMP Expression for Tumor Expansion. Proc Natl Acad Sci USA (2009) 106(30):12530–5. doi: 10.1073/pnas.0804273106 PMC2718387 19617536

[107] Saederup N Cardona AE Croft K Mizutani M Cotleur AC Tsou CL. Selective Chemokine Receptor Usage by Central Nervous System Myeloid Cells in CCR2-Red Fluorescent Protein Knock-in Mice. PloS One (2010) 5(10):e13693. doi: 10.1371/journal.pone.0013693 21060874 PMC2965160

[108] Zhang Y Yu G Chu H Wang X Xiong L Cai G. Macrophage-Associated PGK1 Phosphorylation Promotes Aerobic Glycolysis and Tumorigenesis. Mol Cell (2018) 71(2):201–15.e7. doi: 10.1016/j.molcel.2018.06.023 30029001

[109] Lu J Xu Z Duan H Ji H Zhen Z Li B. Tumor-Associated Macrophage Interleukin-Beta Promotes Glycerol-3-Phosphate Dehydrogenase Activation, Glycolysis and Tumorigenesis in Glioma Cells. Cancer Sci (2020) 111(6):1979–90. doi: 10.1111/cas.14408 PMC7293068 32259365

[110] Carvalho da Fonseca AC Wang H Fan H Chen X Zhang I Zhang L. Increased Expression of Stress Inducible Protein 1 in Glioma-Associated Microglia/Macrophages. J Neuroimmunol (2014) 274(1-2):71–7. doi: 10.1016/j.jneuroim.2014.06.021 PMC4152559 25042352

[111] Wesolowska A Kwiatkowska A Slomnicki L Dembinski M Master A Sliwa M. Microglia-Derived TGF-Beta as an Important Regulator of Glioblastoma Invasion–an Inhibition of TGF-Beta-Dependent Effects by shRNA Against Human TGF-Beta Type II Receptor. Oncogene (2008) 27(7):918–30. doi: 10.1038/sj.onc.1210683 17684491

[112] Wick W Platten M Weller M. Glioma Cell Invasion: Regulation of Metalloproteinase Activity by TGF-Beta. J Neurooncol (2001) 53(2):177–85. doi: 10.1023/A:1012209518843 11716069

[113] Zhu C Mustafa D Zheng PP van der Weiden M Sacchetti A Brandt M. Activation of CECR1 in M2-Like TAMs Promotes Paracrine Stimulation-Mediated Glial Tumor Progression. Neuro Oncol (2017) 19(5):648–59. doi: 10.1093/neuonc/now251 PMC5464467 28453746

[114] Shi Y Ping YF Zhou W He ZC Chen C Bian BS. Tumour-Associated Macrophages Secrete Pleiotrophin to Promote PTPRZ1 Signalling in Glioblastoma Stem Cells for Tumour Growth. Nat Commun (2017) 8:15080. doi: 10.1038/ncomms15080 28569747 PMC5461490

[115] Wang Y Liu T Yang N Xu S Li X Wang D. Hypoxia and Macrophages Promote Glioblastoma Invasion by the CCL4-CCR5 Axis. Oncol Rep (2016) 36(6):3522–8. doi: 10.3892/or.2016.5171 27748906

[116] Yu-Ju Wu C Chen CH Lin CY Feng LY Lin YC Wei KC. CCL5 of Glioma-Associated Microglia/Macrophages Regulates Glioma Migration and Invasion *via* Calcium-Dependent Matrix Metalloproteinase 2. Neuro Oncol (2020) 22(2):253–66. doi: 10.1093/neuonc/noz189 PMC7032635 31593589

[117] Zhang X Chen L Dang WQ Cao MF Xiao JF Lv SQ. CCL8 Secreted by Tumor-Associated Macrophages Promotes Invasion and Stemness of Glioblastoma Cells *via* ERK1/2 Signaling. Lab Invest (2020) 100(4):619–29. doi: 10.1038/s41374-019-0345-3 31748682

[118] Lehnardt S. Innate Immunity and Neuroinflammation in the CNS: The Role of Microglia in Toll-Like Receptor-Mediated Neuronal Injury. Glia (2010) 58(3):253–63. doi: 10.1002/glia.20928 19705460

[119] Vinnakota K Hu F Ku MC Georgieva PB Szulzewsky F Pohlmann A. Toll-Like Receptor 2 Mediates Microglia/Brain Macrophage MT1-MMP Expression and Glioma Expansion. Neuro Oncol (2013) 15(11):1457–68. doi: 10.1093/neuonc/not115 PMC3813418 24014382

[120] Hu F Dzaye O Hahn A Yu Y Scavetta RJ Dittmar G. Glioma-Derived Versican Promotes Tumor Expansion *via* Glioma-Associated Microglial/Macrophages Toll-Like Receptor 2 Signaling. Neuro Oncol (2015) 17(2):200–10. doi: 10.1093/neuonc/nou324 PMC4288527 25452390

[121] Hu F Ku MC Markovic D Dzaye O Lehnardt S Synowitz M. Glioma-Associated Microglial MMP9 Expression Is Upregulated by TLR2 Signaling and Sensitive to Minocycline. Int J Cancer (2014) 135(11):2569–78. doi: 10.1002/ijc.28908 PMC4519695 24752463

[122] Barker N Clevers H. Mining the Wnt Pathway for Cancer Therapeutics. Nat Rev Drug Discov (2006) 5(12):997–1014. doi: 10.1038/nrd2154 17139285

[123] Niehrs C. The Complex World of WNT Receptor Signalling. Nat Rev Mol Cell Biol (2012) 13(12):767–79. doi: 10.1038/nrm3470 23151663

[124] Lee Y Lee JK Ahn SH Lee J Nam DH. WNT Signaling in Glioblastoma and Therapeutic Opportunities. Lab Invest (2016) 96(2):137–50. doi: 10.1038/labinvest.2015.140 26641068

[125] Brennan C Momota H Hambardzumyan D Ozawa T Tandon A Pedraza A. Glioblastoma Subclasses can be Defined by Activity Among Signal Transduction Pathways and Associated Genomic Alterations. PloS One (2009) 4(11):e7752. doi: 10.1371/journal.pone.0007752 19915670 PMC2771920

[126] Gong A Huang S. FoxM1 and Wnt/beta-Catenin Signaling in Glioma Stem Cells. Cancer Res (2012) 72(22):5658–62. doi: 10.1158/0008-5472.CAN-12-0953 PMC3500394 23139209

[127] Matias D Predes D Niemeyer Filho P Lopes MC Abreu JG Lima FRS. Microglia-Glioblastoma Interactions: New Role for Wnt Signaling. Biochim Biophys Acta Rev Cancer (2017) 1868(1):333–40. doi: 10.1016/j.bbcan.2017.05.007 28554667

[128] Azambuja JH Ludwig N Yerneni SS Braganhol E Whiteside TL. Arginase-1+ Exosomes From Reprogrammed Macrophages Promote Glioblastoma Progression. Int J Mol Sci (2020) 21(11):3990. doi: 10.3390/ijms21113990 PMC7312363 32498400

[129] Chen X Zhang L Zhang IY Liang J Wang H Ouyang M. RAGE Expression in Tumor-Associated Macrophages Promotes Angiogenesis in Glioma. Cancer Res (2014) 74(24):7285–97. doi: 10.1158/0008-5472.CAN-14-1240 PMC4268204 25326491

[130] Li Y Guo S Zhao K Conrad C Driescher C Rothbart V. ADAM8 Affects Glioblastoma Progression by Regulating Osteopontin-Mediated Angiogenesis. Biol Chem (2021) 402(2):195–206. doi: 10.1515/hsz-2020-0184 33544472

[131] Zhu C Chrifi I Mustafa D van der Weiden M Leenen PJM Duncker DJ. CECR1-Mediated Cross Talk Between Macrophages and Vascular Mural Cells Promotes Neovascularization in Malignant Glioma. Oncogene (2017) 36(38):5356–68. doi: 10.1038/onc.2017.145 PMC5611481 28534507

[132] Cui X Morales RT Qian W Wang H Gagner JP Dolgalev I. Hacking Macrophage-Associated Immunosuppression for Regulating Glioblastoma Angiogenesis. Biomaterials (2018) 161:164–78. doi: 10.1016/j.biomaterials.2018.01.053 PMC8059366 29421553

[133] Wang Q He Z Huang M Liu T Wang Y Xu H. Vascular Niche IL-6 Induces Alternative Macrophage Activation in Glioblastoma Through HIF-2alpha. Nat Commun (2018) 9(1):559. doi: 10.1038/s41467-018-03050-0 29422647 PMC5805734

[134] Blank A Kremenetskaia I Urbantat RM Acker G Turkowski K Radke J. Microglia/macrophages Express Alternative Proangiogenic Factors Depending on Granulocyte Content in Human Glioblastoma. J Pathol (2021) 253(2):160–73. doi: 10.1002/path.5569 33044746

[135] Hoelzinger DB Demuth T Berens ME. Autocrine Factors That Sustain Glioma Invasion and Paracrine Biology in the Brain Microenvironment. J Natl Cancer Inst (2007) 99(21):1583–93. doi: 10.1093/jnci/djm187 17971532

[136] Pyonteck SM Akkari L Schuhmacher AJ Bowman RL Sevenich L Quail DF. CSF-1R Inhibition Alters Macrophage Polarization and Blocks Glioma Progression. Nat Med (2013) 19(10):1264–72. doi: 10.1038/nm.3337 PMC3840724 24056773

[137] Quail DF Bowman RL Akkari L Quick ML Schuhmacher AJ Huse JT. The Tumor Microenvironment Underlies Acquired Resistance to CSF-1R Inhibition in Gliomas. Science (2016) 352(6288):aad3018. doi: 10.1126/science.aad3018 27199435 PMC5450629

[138] Butowski N Colman H De Groot JF Omuro AM Nayak L Wen PY. Orally Administered Colony Stimulating Factor 1 Receptor Inhibitor PLX3397 in Recurrent Glioblastoma: An Ivy Foundation Early Phase Clinical Trials Consortium Phase II Study. Neuro Oncol (2016) 18(4):557–64. doi: 10.1093/neuonc/nov245 PMC4799682 26449250

[139] Escobar G Moi D Ranghetti A Ozkal-Baydin P Squadrito ML Kajaste-Rudnitski A. Genetic Engineering of Hematopoiesis for Targeted IFN-Alpha Delivery Inhibits Breast Cancer Progression. Sci Transl Med (2014) 6(217):217ra3. doi: 10.1126/scitranslmed.3006353 24382895

[140] Gschwandtner M Derler R Midwood KS. More Than Just Attractive: How CCL2 Influences Myeloid Cell Behavior Beyond Chemotaxis. Front Immunol (2019) 10:2759. doi: 10.3389/fimmu.2019.02759 31921102 PMC6923224

[141] Peng SB Zhang X Paul D Kays LM Gough W Stewart J. Identification of LY2510924, a Novel Cyclic Peptide CXCR4 Antagonist That Exhibits Antitumor Activities in Solid Tumor and Breast Cancer Metastatic Models. Mol Cancer Ther (2015) 14(2):480–90. doi: 10.1158/1535-7163.MCT-14-0850 25504752

[142] Mercurio L Ajmone-Cat MA Cecchetti S Ricci A Bozzuto G Molinari A. Targeting CXCR4 by a Selective Peptide Antagonist Modulates Tumor Microenvironment and Microglia Reactivity in a Human Glioblastoma Model. J Exp Clin Cancer Res (2016) 35:55. doi: 10.1186/s13046-016-0326-y 27015814 PMC4807593

[143] Thomas RP Nagpal S Iv M Soltys SG Bertrand S Pelpola JS. Macrophage Exclusion After Radiation Therapy (MERT): A First in Human Phase I/II Trial Using a CXCR4 Inhibitor in Glioblastoma. Clin Cancer Res (2019) 25(23):6948–57. doi: 10.1158/1078-0432.CCR-19-1421 PMC6891194 31537527

